# Supplementary material for: Multiple Reinventions of Mating-type Switching during Budding Yeast Evolution
Source: Curr Biol. 2019 Aug 5;29(15):2555–2562.e8. doi: 10.1016/j.cub.2019.06.056 (PMC6692504; doi:10.1016/j.cub.2019.06.056)
Supplement: Document S1. Figure S1 [file mmc1.pdf]

Current Biology, Volume *29*

# Supplemental Information

# Multiple Reinventions of Mating-type Switching

# during Budding Yeast Evolution

**Tadeusz Krassowski, Jacek Kominek, Xing-Xing Shen, Dana A. Opulente, Xiaofan Zhou, Antonis Rokas, Chris Todd Hittinger, and Kenneth H. Wolfe**

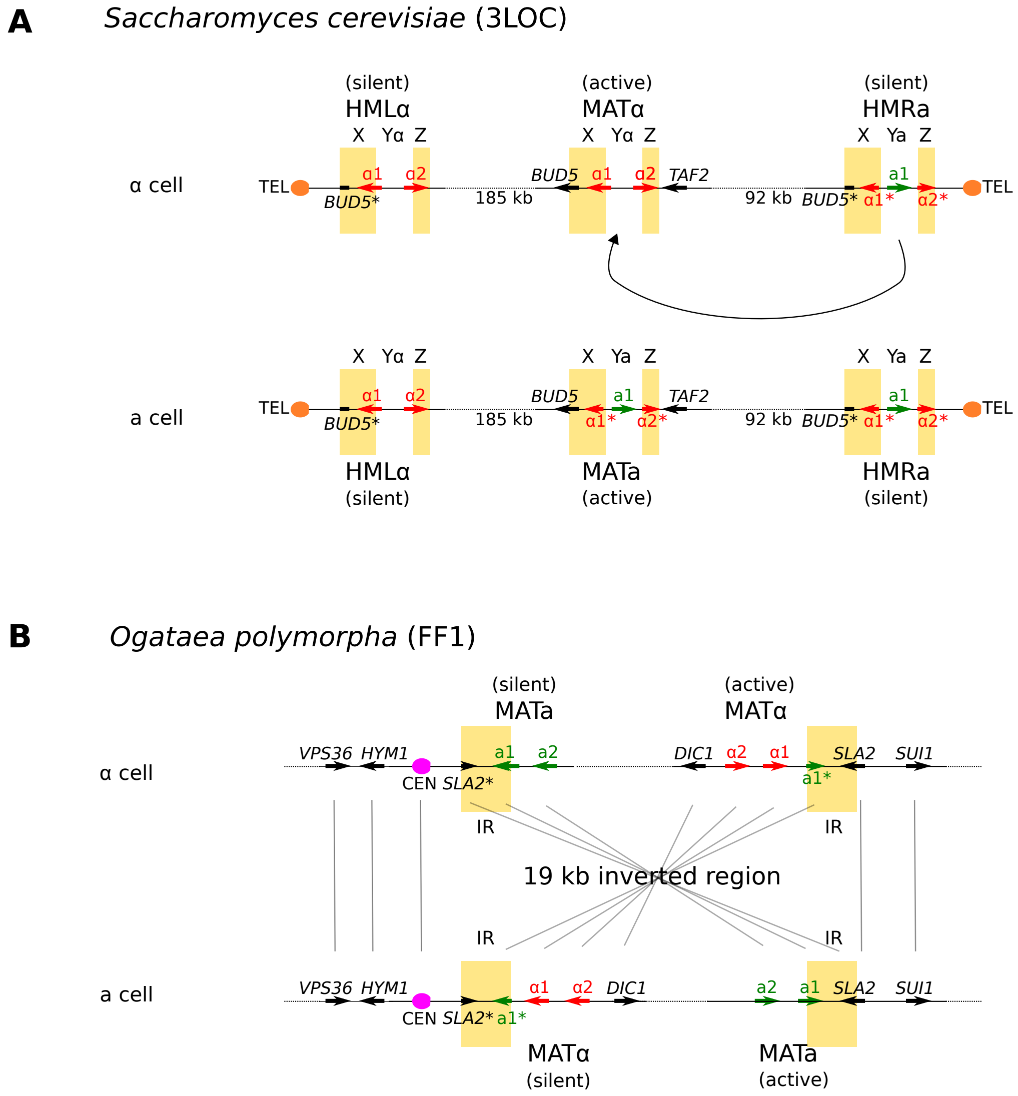


**Figure S1. Genomic organization of MAT loci and mating-type switching in *Saccharomyces cerevisiae* and *Ogataea polymorpha*. Related to Figure 1.**

Yellow boxes indicate repeats.
