# Supplementary figures and images for: Multiple Reinventions of Mating-type Switching during Budding Yeast Evolution
Source: Curr Biol. 2019 Aug 5;29(15):2555–2562.e8. doi: 10.1016/j.cub.2019.06.056 (PMC6692504; doi:10.1016/j.cub.2019.06.056)

Tree scale: 1 

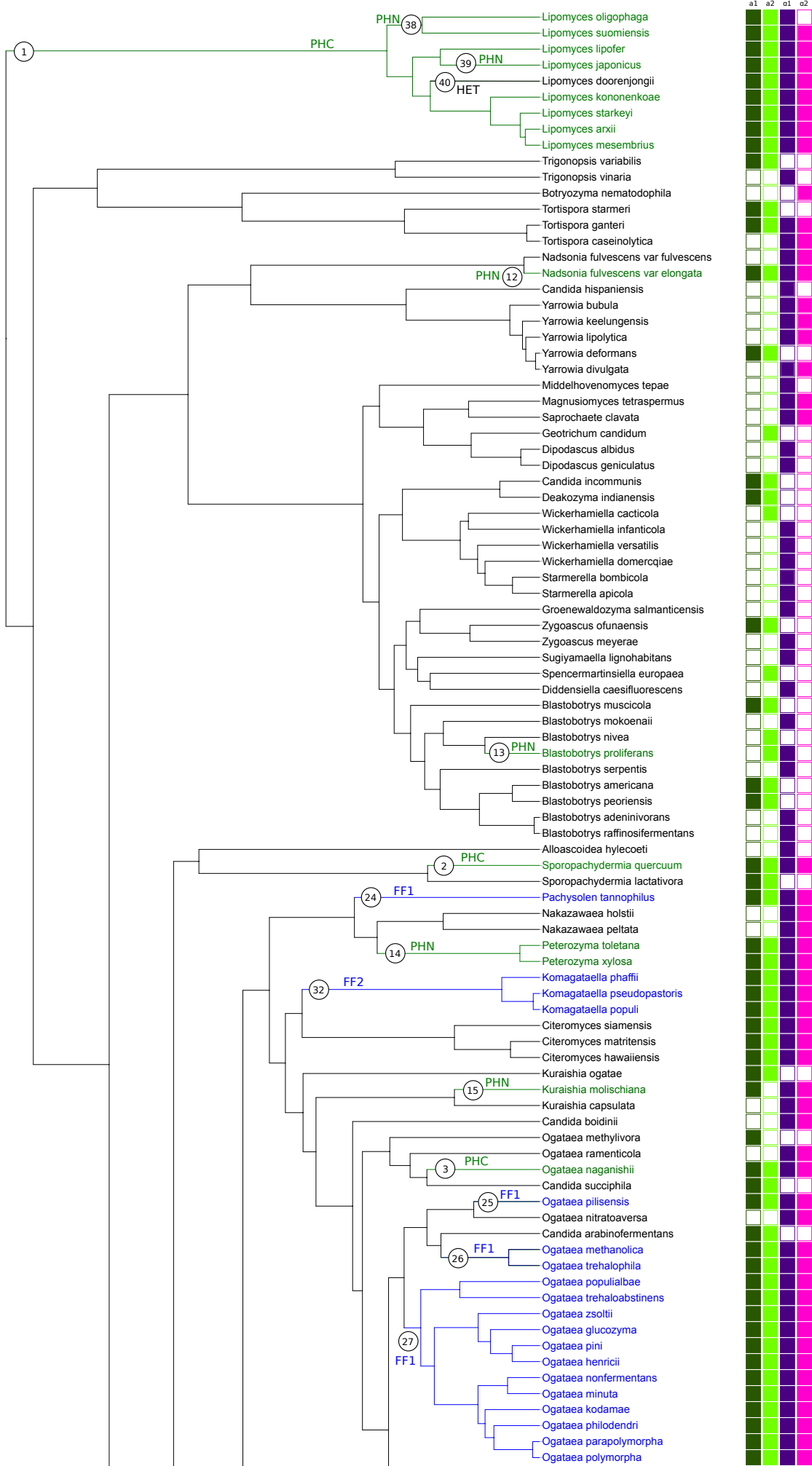

Tree scale: 1

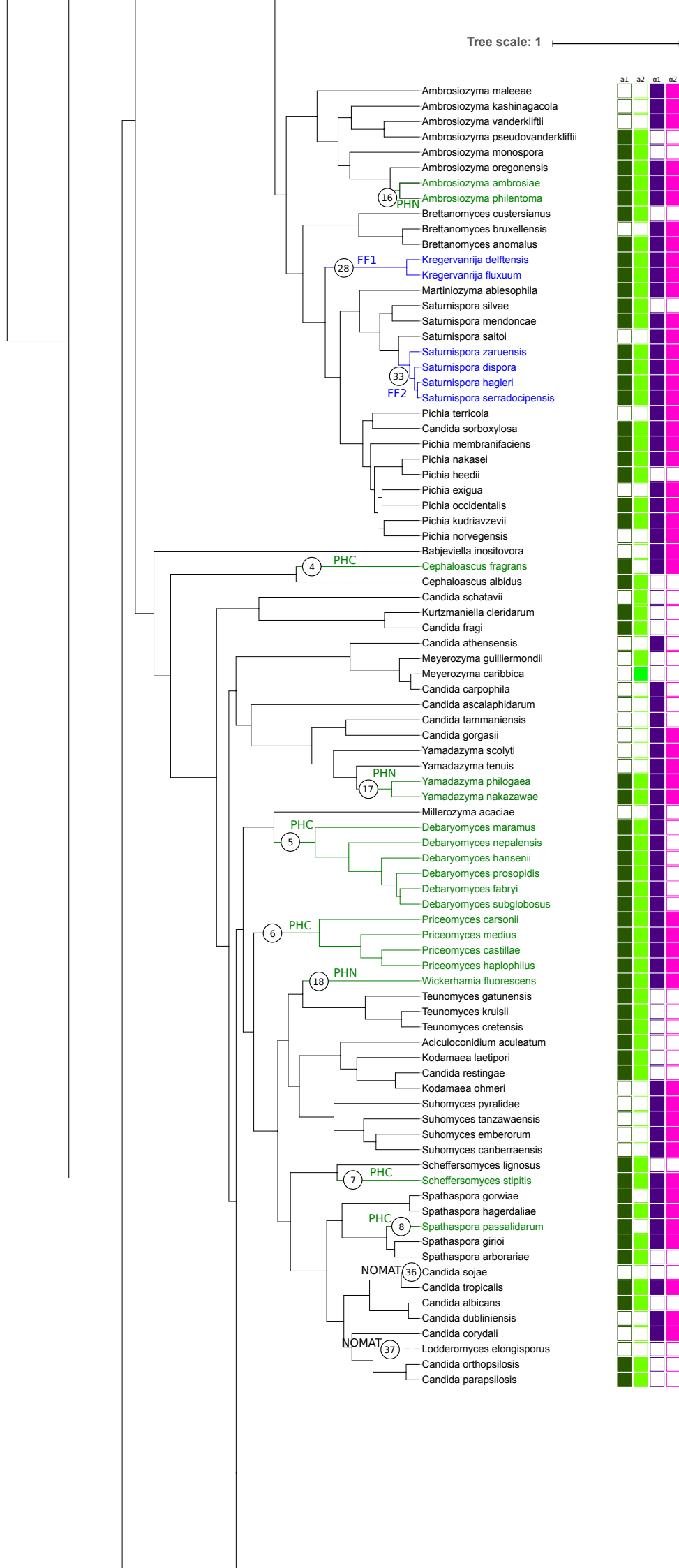

Tree scale: 1

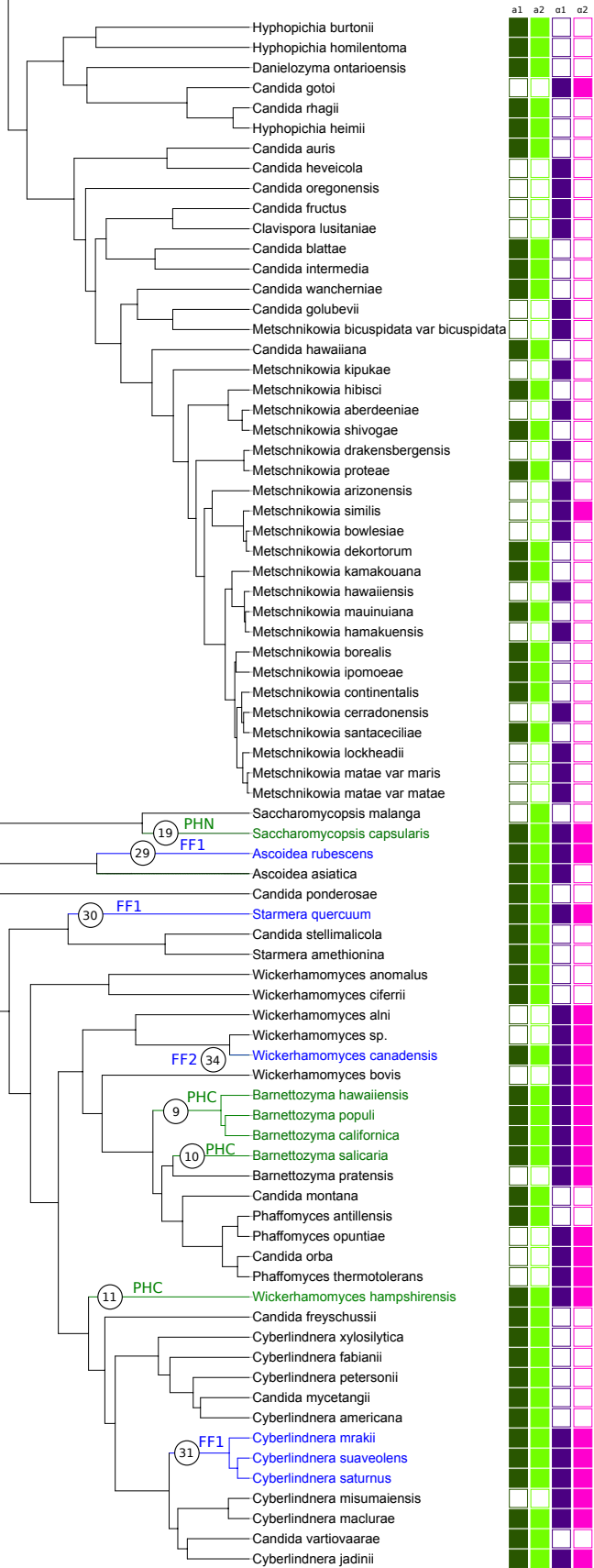

Tree scale: 1

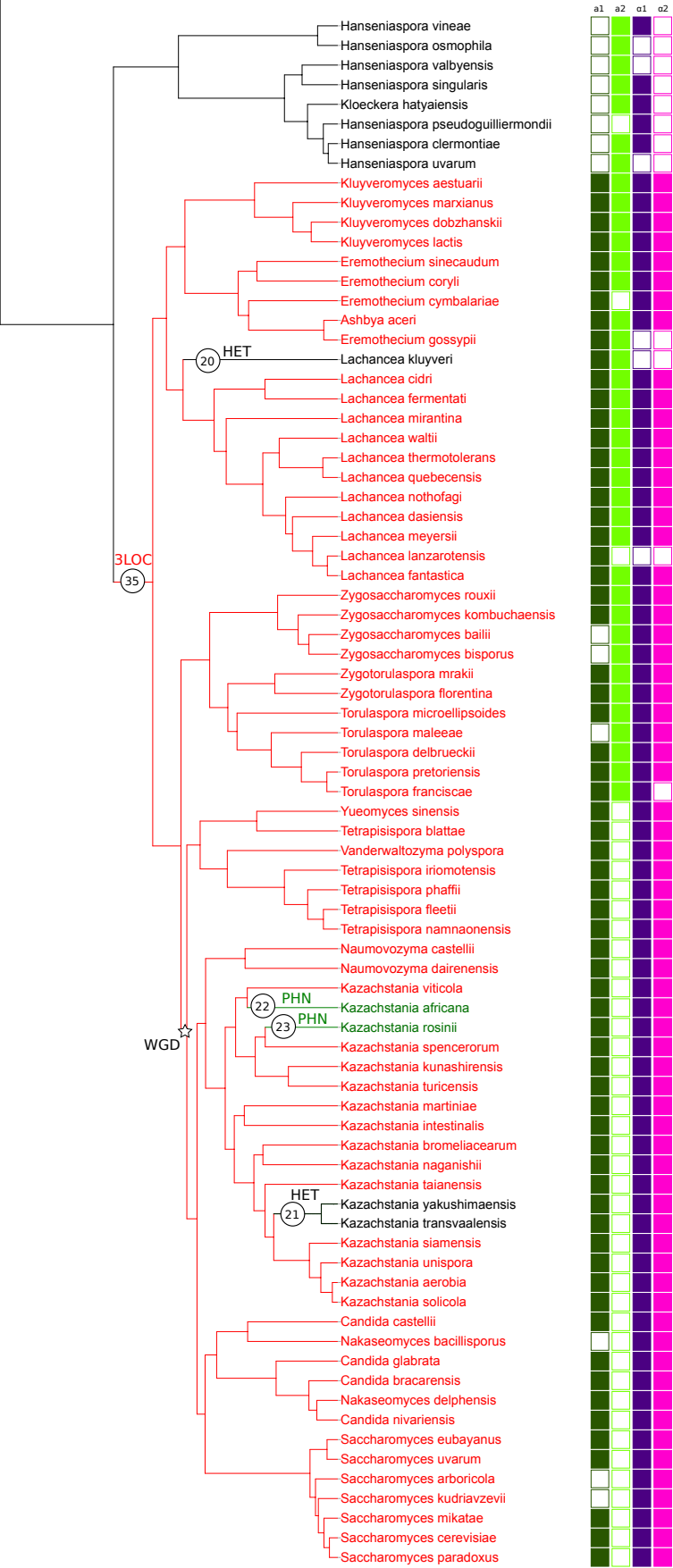

Supplement: Data S1. MAT Gene Content and Inferred Transitions of Mating Compatibility Systems in 332 Budding Yeast Species, Related to Figure 2 and STAR Methods — Colored boxes on the right indicate the presence of the MAT genes a1 (dark green), a2 (light green), α1 (purple) and α2 (pink), with white boxes indicating absence. Branch colors indicate inferred heterothallism (HET; black), primary homothallism (PHC or PHN; green), secondary homothallism by flip/flop mating-type switching (FF1 or FF2; blue), or secondary homothallism by a three-locus switching system (3LOC; red). Circled numbers indicate inferred transitions between mating compatibility systems: HET → PHC (numbers 1- 11); HET → PHN (12-19); 3LOC → HET (20-21); 3LOC → PHN (22-23); HET → FF1 (24-31); HET → FF2 (32-34); HET → 3LOC (35); HET → NOMAT (36-37); PHC → PHN (38-39); PHC → HET (40). The star indicates the point at which whole genome duplication happened. [file mmc2.pdf]
