# Supplementary material for: Multiple Reinventions of Mating-type Switching during Budding Yeast Evolution
Source: Curr Biol. 2019 Aug 5;29(15):2555–2562.e8. doi: 10.1016/j.cub.2019.06.056 (PMC6692504; doi:10.1016/j.cub.2019.06.056)
Supplement: Data S2. Synteny Arrangements in Chosen Species, Related to Figure 2 and STAR Methods — (A) MAT locus arrangement and synteny relationship between Ascoidea asiatica (HET, two alleles shown) and Ascoidea rubescens (FF1). A. asiatica has two divergent copies of STE20 and AGE1 located within the a and α alleles of its MAT locus. In A. rubescens, the MATa and MATα genes are separated by 44 kb of noncoding DNA. Spirals denote inverted genes. Phylogenetic tree of AGE1 sequences from Ascoidea and related species. The single AGE1 gene of A. rubescens groups with A. asiatica AGE1a, with A. asiatica AGE1α outside. Phylogenetic tree of STE20 and CLA4 PAK kinases from Ascoidea and related species. A. rubescens has no STE20 gene, but has a duplication of CLA4. The trees in (B) and (C) were constructed from amino acid sequences using PhyML with MUSCLE alignment and Gblocks filtering, as implemented in Seaview version 4.5.0 with Seaview default parameters for all programs. Bootstrap values from 100 replicates are indicated. (B) MAT locus arrangement and synteny relationship between Pachysolen tannophilus (FF1), Peterozyma xylosa (PHN), and Peterozyma toletana (PHN). (C) MAT locus organization in three species inferred to switch mating types by inversion using one IR (FF1 species). For each of these FF1 species, a DISCOVAR assembly of the genome indicated the presence of an IR of undetermined size (see STAR Methods). The species are Cyberlindnera saturnus (FF1) compared to Cyberlindnera jadinii (HET), Starmera quercuum (FF1) and Kregervanrija fluxuum (FF1). (D) MAT locus arrangement and synteny relationship between Wickerhamomyces sp. NRRL YB-2243 (HET) and Wickerhamomyces canadensis (FF2). In W. canadensis, the MATa genes are in the middle of a 612-kb scaffold (NODE_1), and the MATα genes are on a 7-kb scaffold (NODE_70). (E) MAT locus arrangement and synteny relationship between Nadsonia starkeyi-henricii (HET), Nadsonia fulvescens var. fulvescens (HET), and Nadsonia fulvescens var. elongata (PHN). The MATa genes of N. fulvescens var. elongata have been gained by in [file mmc3.pdf]

A

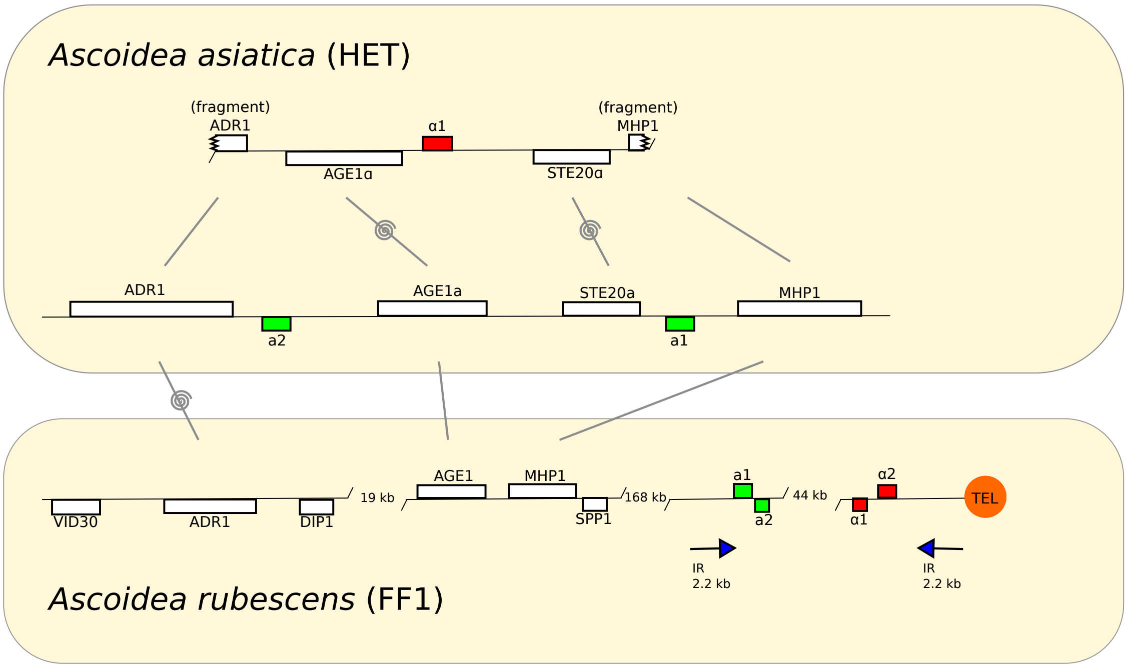
Ascoidea asiatica (HET)
(fragment)
ADR1
α1
(fragment)
MHP1
AGE1α
STE20α
ADR1
AGE1a
STE20a
MHP1
a2
a1
AGE1
MHP1
a1
α2
VID30
ADR1
DIP1
19 kb
SPP1
168 kb
a2
44 kb
α1
TEL
IR
2.2 kb
IR
2.2 kb
Ascoidea rubescens (FF1)

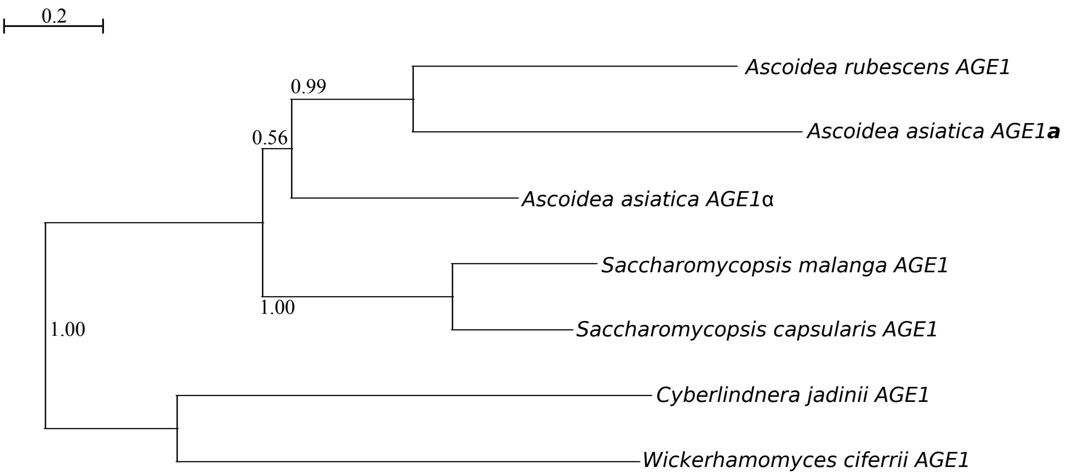
0.2
Ascoidea rubescens AGE1
0.99
Ascoidea asiatica AGE1**a**
0.56
Ascoidea asiatica AGE1α
Saccharomycopsis malanga AGE1
1.00
Saccharomycopsis capsularis AGE1
1.00
Cyberlindnera jadinii AGE1
Wickerhamomyces ciferrii AGE1

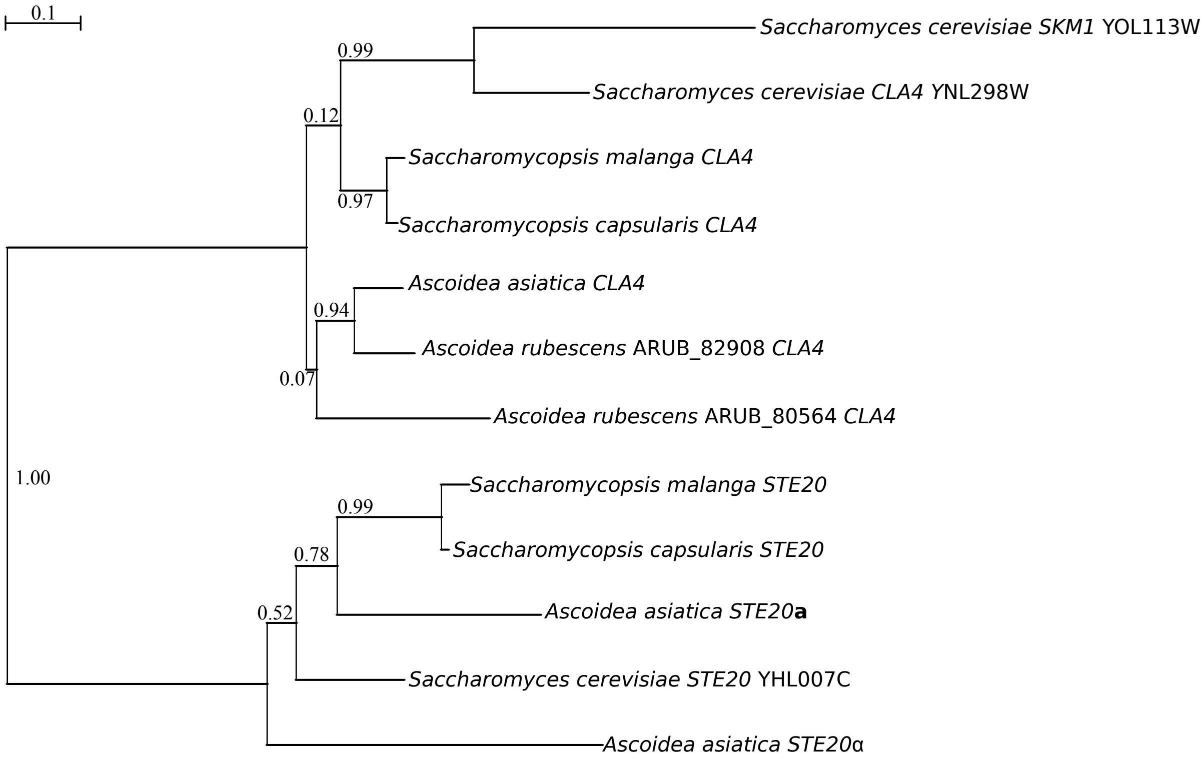
0.1
Saccharomyces cerevisiae SKM1 YOL113W
0.99
Saccharomyces cerevisiae CLA4 YNL298W
0.12
Saccharomycopsis malanga CLA4
0.97
Saccharomycopsis capsularis CLA4
Ascoidea asiatica CLA4
0.94
Ascoidea rubescens ARUB_82908 CLA4
0.07
Ascoidea rubescens ARUB_80564 CLA4
1.00
Saccharomycopsis malanga STE20
0.99
Saccharomycopsis capsularis STE20
0.78
Ascoidea asiatica STE20**a**
0.52
Saccharomyces cerevisiae STE20 YHL007C
Ascoidea asiatica STE20α

B

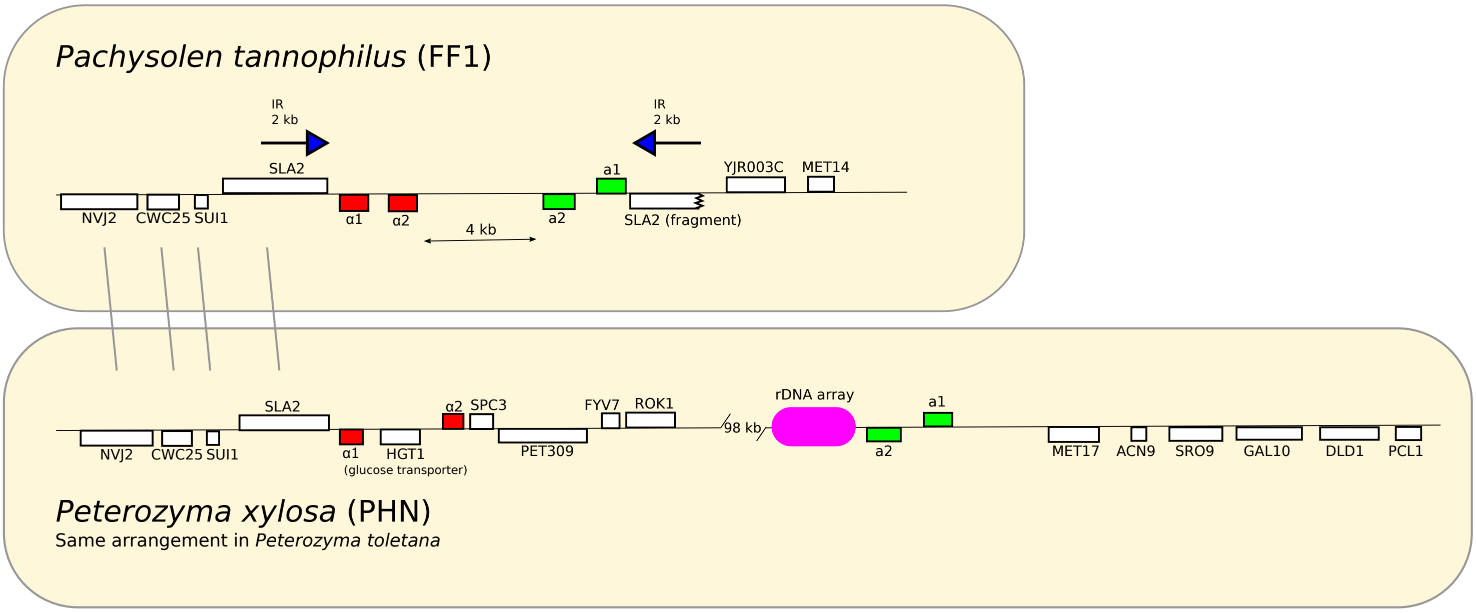
Pachysolen tannophilus (FF1)
IR
2 kb
IR
2 kb
SLA2
a1
YJR003C
MET14
NVJ2
CWC25
SUI1
α1
α2
a2
SLA2 (fragment)
4 kb
SLA2
α2
SPC3
FYV7
ROK1
rDNA array
a1
98 kb
NVJ2
CWC25
SUI1
α1
HGT1
(glucose transporter)
PET309
a2
MET17
ACN9
SRO9
GAL10
DLD1
PCL1
Peterozyma xylosa (PHN)
Same arrangement in Peterozyma toletana

C

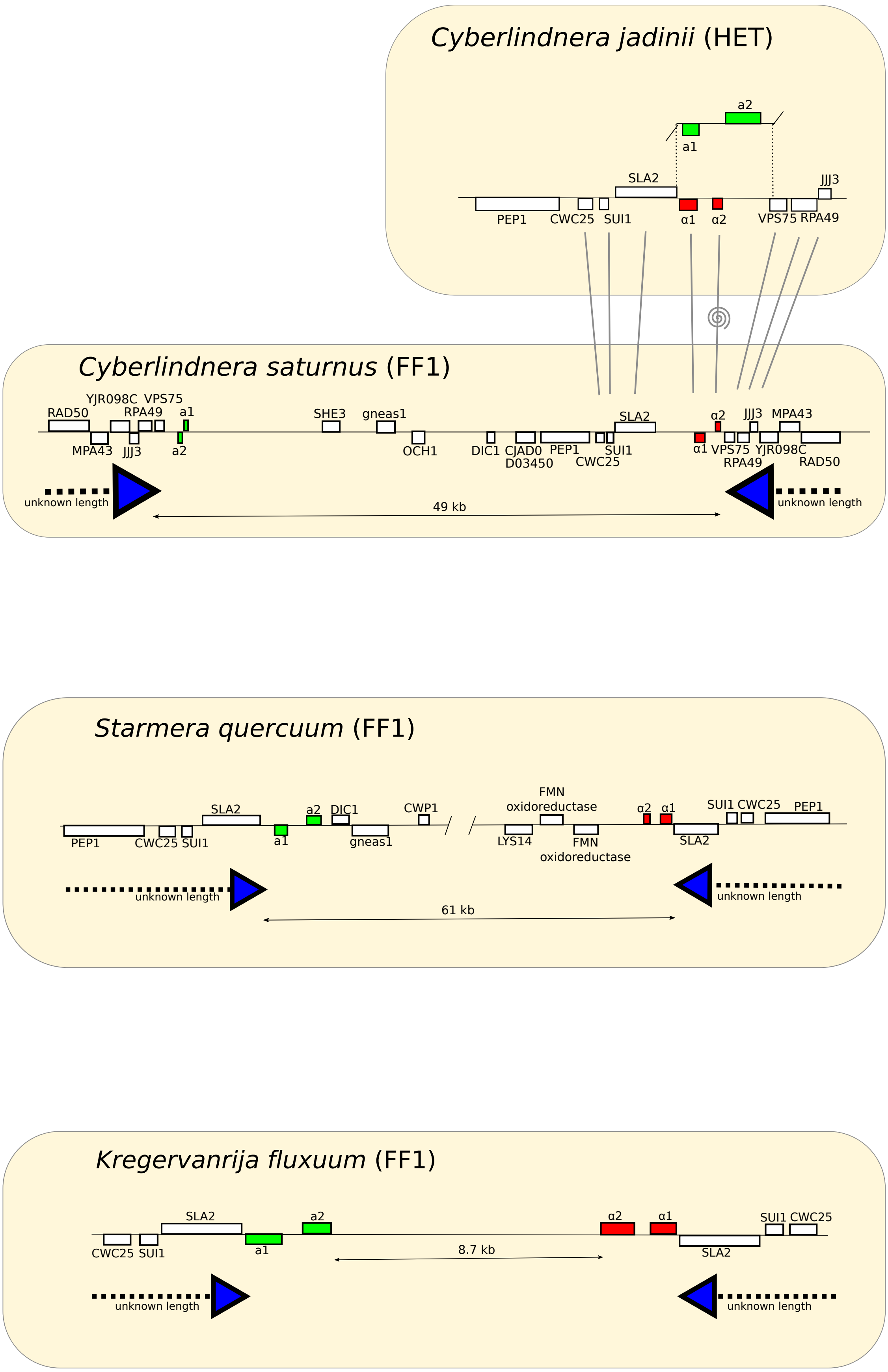

Cyberlindnera jadinii (HET)
a2
a1
SLA2
JJJ3
PEP1
CWC25
SUI1
α1
α2
VPS75
RPA49
Cyberlindnera saturnus (FF1)
YJR098C
VPS75
RAD50
RPA49
a1
SHE3
gneas1
SLA2
α2
JJJ3
MPA43
MPA43
JJJ3
a2
OCH1
DIC1
CJAD0
D03450
PEP1
SUI1
CWC25
α1
VPS75
YJR098C
RPA49
RAD50
unknown length
49 kb
unknown length
Starmera quercuum (FF1)
FMN
oxidoreductase
SLA2
a2
DIC1
CWP1
α2
α1
SUI1
CWC25
PEP1
PEP1
CWC25
SUI1
a1
gneas1
LYS14
FMN
oxidoreductase
SLA2
unknown length
61 kb
unknown length
Kregervanrija fluxuum (FF1)
SLA2
a2
α2
α1
SUI1
CWC25
CWC25
SUI1
a1
8.7 kb
SLA2
unknown length
unknown length

D

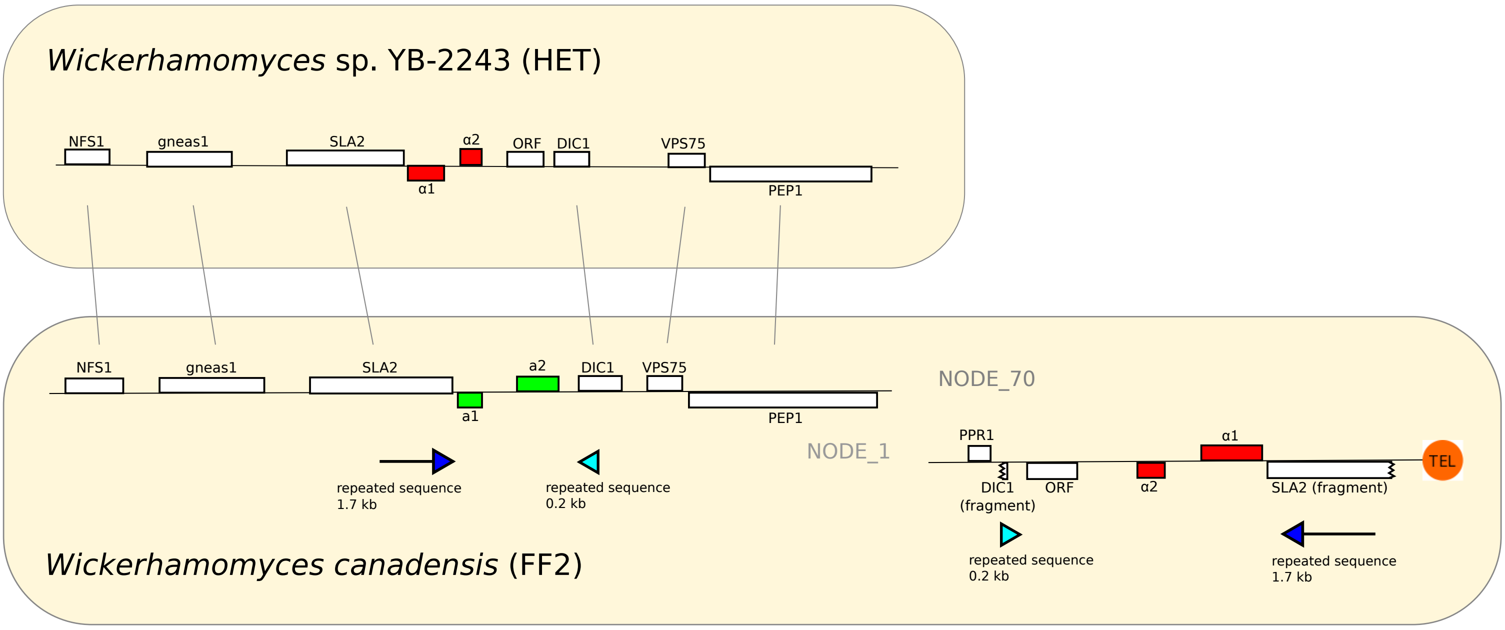

Wickerhamomyces sp. YB-2243 (HET)
NFS1
gneas1
SLA2
α1
α2
ORF
DIC1
VPS75
PEP1
NFS1
gneas1
SLA2
a1
a2
DIC1
VPS75
PEP1
NODE_70
NODE_1
PPR1
DIC1 (fragment)
ORF
α2
α1
SLA2 (fragment)
TEL
repeated sequence 1.7 kb
repeated sequence 0.2 kb
repeated sequence 0.2 kb
repeated sequence 1.7 kb
Wickerhamomyces canadensis (FF2)

E

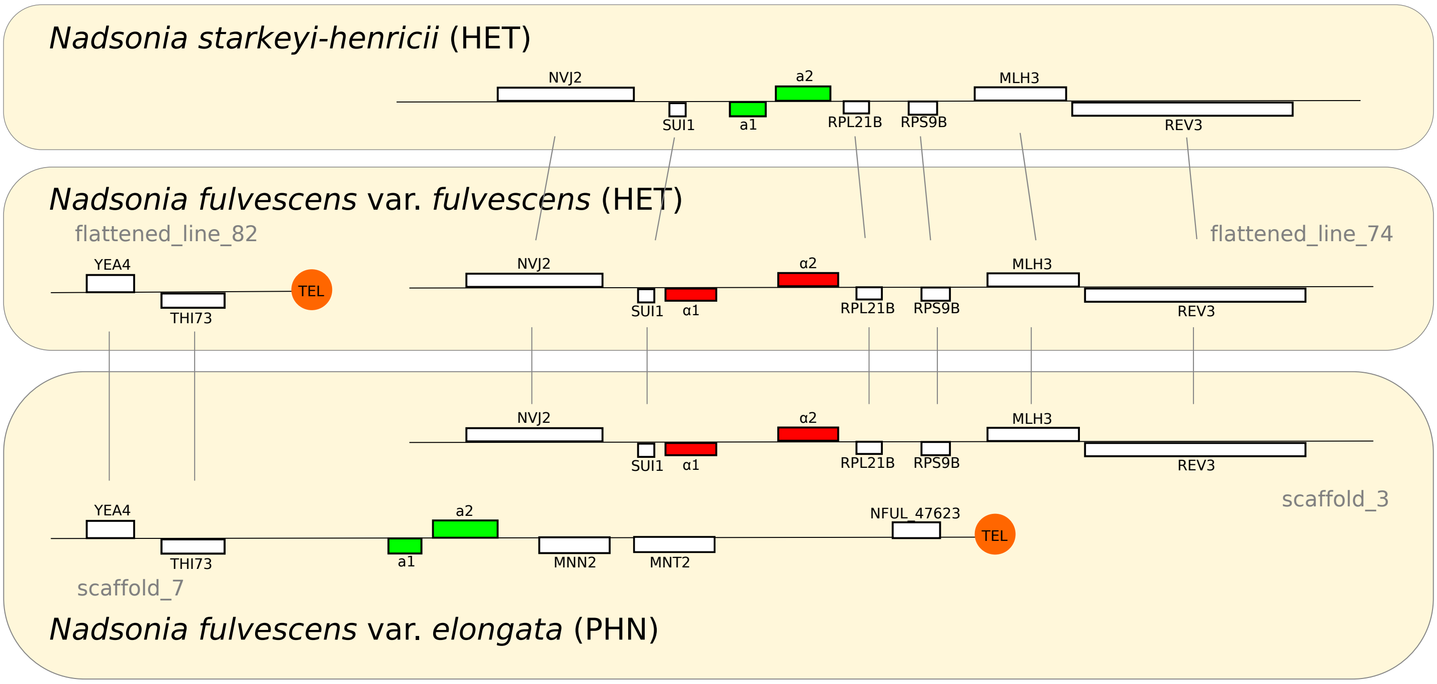
Nadsonia starkeyi-henricii (HET)
NVJ2
a2
MLH3
SUI1
a1
RPL21B
RPS9B
REV3
Nadsonia fulvescens var. fulvescens (HET)
flattened_line_82
flattened_line_74
YEA4
THI73
TEL
NVJ2
α2
MLH3
SUI1
α1
RPL21B
RPS9B
REV3
NVJ2
α2
MLH3
SUI1
α1
RPL21B
RPS9B
REV3
scaffold_3
YEA4
a2
NFUL_47623
TEL
THI73
a1
MNN2
MNT2
scaffold_7
Nadsonia fulvescens var. elongata (PHN)

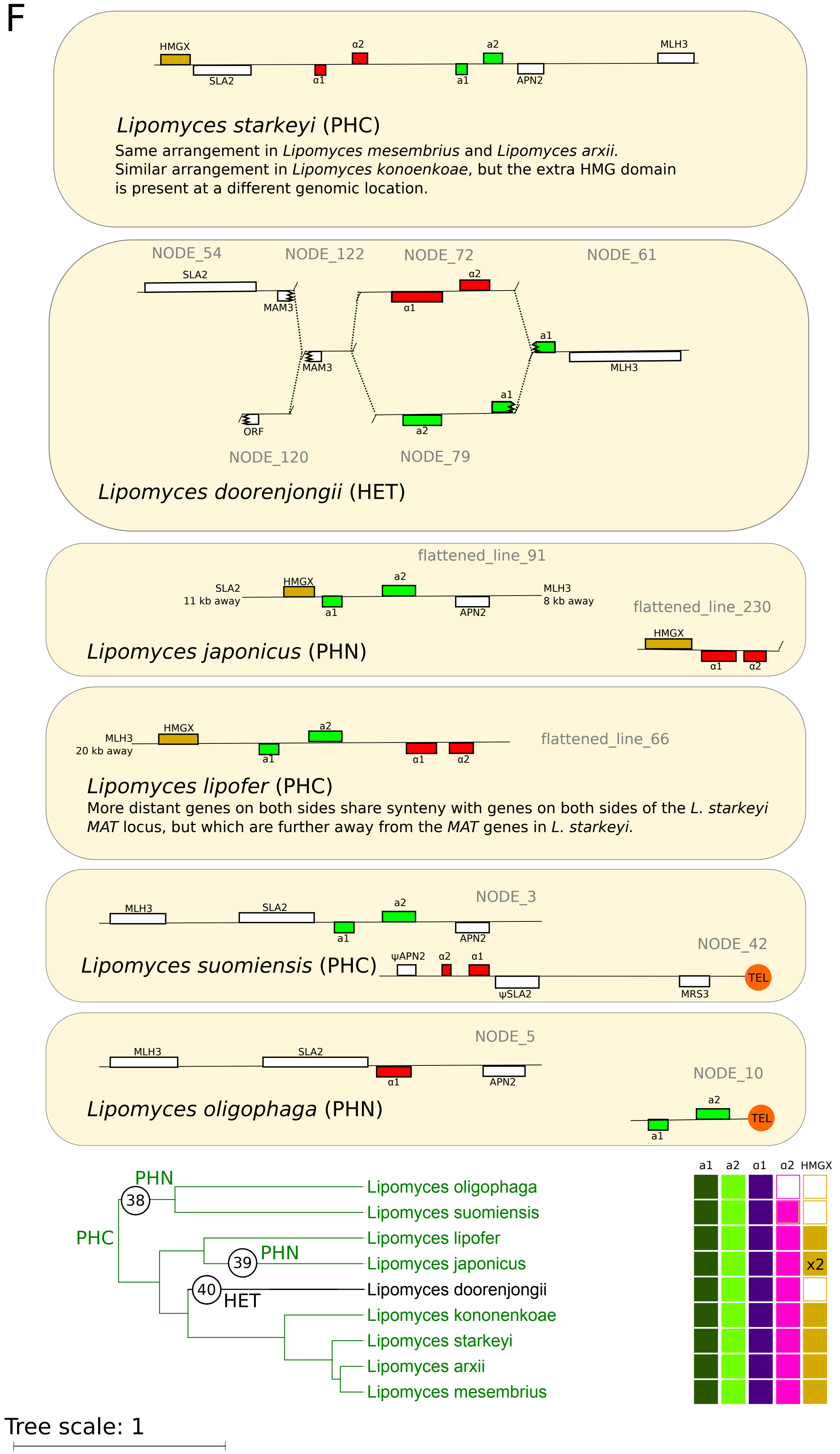
F
HMGX
SLA2
α1
α2
a1
a2
APN2
MLH3
Lipomyces starkeyi (PHC)
Same arrangement in Lipomyces mesembrius and Lipomyces arxii.
Similar arrangement in Lipomyces konoenkoae, but the extra HMG domain is present at a different genomic location.
NODE_54
NODE_122
NODE_72
NODE_61
SLA2
MAM3
α2
α1
a1
MLH3
MAM3
a1
ORF
a2
NODE_120
NODE_79
Lipomyces doorenjongii (HET)
flattened_line_91
SLA2
11 kb away
HMGX
a1
a2
APN2
MLH3
8 kb away
flattened_line_230
HMGX
α1
α2
Lipomyces japonicus (PHN)
MLH3
20 kb away
HMGX
a1
a2
α1
α2
flattened_line_66
Lipomyces lipofer (PHC)
More distant genes on both sides share synteny with genes on both sides of the L. starkeyi MAT locus, but which are further away from the MAT genes in L. starkeyi.
NODE_3
MLH3
SLA2
a1
a2
APN2
NODE_42
Lipomyces suomiensis (PHC)
ψAPN2
α2
α1
ψSLA2
MRS3
TEL
NODE_5
MLH3
SLA2
α1
APN2
NODE_10
Lipomyces oligophaga (PHN)
a1
a2
TEL
PHN
38
PHC
PHN
39
40
HET
Lipomyces oligophaga
Lipomyces suomiensis
Lipomyces lipofer
Lipomyces japonicus
Lipomyces doorenjongii
Lipomyces kononenkoae
Lipomyces starkeyi
Lipomyces arxii
Lipomyces mesembrius
a1
a2
α1
α2
HMGX
x2
Tree scale: 1
