# Supplementary material for: Multiple Reinventions of Mating-type Switching during Budding Yeast Evolution
Source: Curr Biol. 2019 Aug 5;29(15):2555–2562.e8. doi: 10.1016/j.cub.2019.06.056 (PMC6692504; doi:10.1016/j.cub.2019.06.056)
Supplement: Document S2. Article plus Supplemental Information [file mmc4.pdf]

Report

Current Biology

Multiple Reinventions of Mating-type Switching during Budding Yeast Evolution

Highlights

- Mating-type switching by flip-flopping arose at least 10 separate times
- Mating-type switching by copy-and-paste arose only once in budding yeasts
- Transitions toward homothallism outnumber transitions away from homothallism
- Heterothallic species can become homothallic by DNA introgression

Authors

Tadeusz Krassowski, Jacek Kominek, Xing-Xing Shen, ..., Antonis Rokas, Chris Todd Hittinger, Kenneth H. Wolfe

Correspondence

kenneth.wolfe@ucd.ie

In Brief

Krassowski et al. find that mating-type switching originated at least 11 times independently during the evolution of budding yeasts, pointing to the adaptive value of self-fertility to unicellular fungi.

Krassowski et al., 2019, Current Biology *29*, 2555–2562
August 5, 2019 © 2019 The Authors. Published by Elsevier Ltd.
https://doi.org/10.1016/j.cub.2019.06.056

Current Biology
Report

CellPress

Multiple Reinventions of Mating-type Switching during Budding Yeast Evolution

Tadeusz Krassowski,[1,2,3] Jacek Kominek,[2,3] Xing-Xing Shen,[4] Dana A. Opulente,[2,3] Xiaofan Zhou,[4] Antonis Rokas,[4] Chris Todd Hittinger,[2,3] and Kenneth H. Wolfe[1,5,*]
[1]Conway Institute and School of Medicine, University College Dublin, Dublin 4, Ireland
[2]Laboratory of Genetics, Genome Center of Wisconsin, Wisconsin Energy Institute, J.F. Crow Institute for the Study of Evolution, University of Wisconsin-Madison, Madison, WI 53706, USA
[3]DOE Great Lakes Bioenergy Research Center, University of Wisconsin-Madison, Madison, WI 53706, USA
[4]Department of Biological Sciences, Vanderbilt University, Nashville, TN 37235, USA
[5]Lead Contact
*Correspondence: kenneth.wolfe@ucd.ie
https://doi.org/10.1016/j.cub.2019.06.056

SUMMARY

Cell type in budding yeasts is determined by the genotype at the mating-type (*MAT*) locus, but yeast species differ widely in their mating compatibility systems and life cycles. Among sexual yeasts, heterothallic species are those in which haploid strains fall into two distinct and stable mating types (*MATa* and *MAT*α), whereas homothallic species are those that can switch mating types or that appear not to have distinct mating types [1, 2]. The evolutionary history of these mating compatibility systems is uncertain, particularly regarding the number and direction of transitions between homothallism and heterothallism, and regarding whether the process of mating-type switching had a single origin [3–5]. Here, we inferred the mating compatibility systems of 332 budding yeast species from their genome sequences. By reference to a robust phylogenomic tree [6], we detected evolutionary transitions between heterothallism and homothallism, and among different forms of homothallism. We find that mating-type switching has arisen independently at least 11 times during yeast evolution and that transitions from heterothallism to homothallism greatly outnumber transitions in the opposite direction (31 versus 3). Although the 3-locus *MAT-HML-HMR* mechanism of mating-type switching as seen in *Saccharomyces cerevisiae* had a single evolutionary origin in budding yeasts, simpler "flip/flop" mechanisms of switching evolved separately in at least 10 other groups of yeasts. These results point to the adaptive value of homothallism and mating-type switching to unicellular fungi.

RESULTS AND DISCUSSION

Inferring Mating Compatibility Systems from Genome Sequences

Shen, Opulente, Kominek, Zhou, et al. [6] recently reported a phylogeny of budding yeasts, based on the genome sequences of 332 species. We analyzed these sequences to infer the "mating compatibility system" of each species, by which we mean its thallism state (i.e., whether it is heterothallic or homothallic) and, for each homothallic species, the molecular basis of its homothallism [2–5, 7].

To identify *MAT*-like loci, we searched for genes coding for the four canonical *MAT* proteins (a1, a2, α1, and α2) in a reference genome sequence (one strain) from each species [6], by using automated TBLASTN searches with a diverse set of *MAT* protein sequences as queries. Genomic regions containing *MAT* genes were then examined by eye to validate and classify the loci. We classified each species as having one of seven possible mating compatibility systems (Figure 1) based on its genome's content of *MAT* genes and the presence or absence of repeated sequences near them, as summarized below. Detailed descriptions of each species' status are given in STAR Methods.

HET

In heterothallic species, strains occur as two different mating types, and the mating types are stable. We classified species as heterothallic (HET; Figure 1A) if their genome sequences contain only *MATa* genes or only *MAT*α genes (presumed heterothallic haploids) or if they contain both *MATa* and *MAT*α genes on separate contigs that appear to be allelic (presumed heterothallic diploids).

In homothallic species, strains do not fall into two distinct mating types. Instead, any strain can mate with any other strain. Two major forms of homothallism are recognized—primary and secondary—with mating-type switching occurring only in secondary homothallics [2, 3, 8]. In primary homothallic species, it is thought that any cell can mate with any other cell, though this has not been investigated in detail [5]. In secondary homothallic species, cells have distinct mating types and mating always occurs between a *MATa* cell and a *MAT*α cell, but strains do not maintain stable mating types because cells can switch their mating type. We classified species into three distinct genomic categories of secondary homothallism (3LOC, FF1, and FF2) and two distinct genomic categories of primary homothallism (PHC and PHN), as explained below, based on their inferred molecular mechanisms. These terms describe categories of genomic organization, not phylogenetic groups.

3LOC

Among the secondary homothallics, we refer to species such as *Saccharomyces cerevisiae* as having a three-locus system

This is an open access article under the CC BY license (http://creativecommons.org/licenses/by/4.0/).

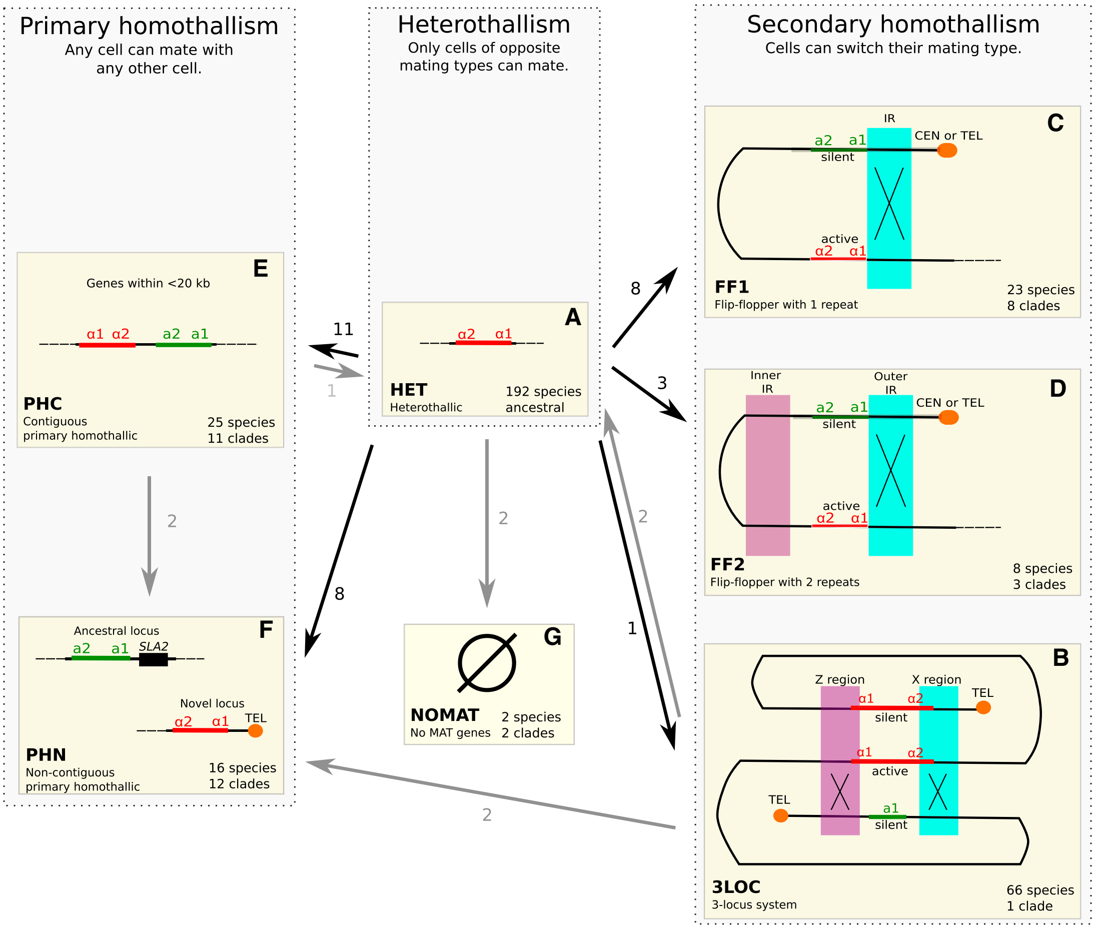

Figure 1. Seven Categories of Mating Compatibility Systems in Budding Yeast Species

The arrows show the inferred numbers of evolutionary transitions between different systems.

(A) Heterothallism. Strains of a heterothallic species are either haploid *MAT*α (as shown), haploid *MAT*a, or diploid *MAT*a/α.

(B–D) Three systems of secondary homothallism: 3LOC (B), FF1 (C), and FF2 (D). Purple and blue shading indicates DNA sequences that form repeats (IRs, Z and X regions) that participate in DNA exchanges (X symbols) during mating-type switching. FF1 and FF2 are defined by the presence of 1 or 2 sets of IRs, respectively. The locations of the elements that repress transcription (centromeres or telomeres) relative to the IRs can vary among species.

(E and F) Two systems of primary homothallism: PHC (E) and PHN (F). In PHC, the two types of *MAT* genes are contiguous on the chromosome. In PHN, they are non-contiguous. The cartoon shows a typical PHN arrangement, where one *MAT* locus is at the ancestral position beside *SLA2*, and a second, novel, *MAT* locus is near a telomere.

(G) Species with no evident *MAT* genes were classified as NOMAT.

See also Figure S1.

(3LOC; Figures 1B and S1A). They have an active *MAT* locus and two or more silent loci (called *HML* and *HMR* in *S. cerevisiae*) that contain non-expressed a and α sequence information [9, 10]. They switch mating types by a copy-and-paste mechanism, copying DNA from the silent loci and pasting it into the *MAT* locus. Exchange of DNA between *MAT* and *HML/HMR* is facilitated by DNA repeat sequences called X and Z that flank these three loci (Figure 1B). HO endonuclease, which cleaves the *MAT* locus to initiate mating-type switching in *S. cerevisiae* [10], has a narrow phylogenetic distribution and is only present in a subset of the genera that use the 3LOC system [5]. Similarly, the *KAT1* and "α3" genes that cleave the *MAT* locus in the 3LOC system of *Kluyveromyces* species are also phylogenetically restricted to that genus [11, 12].

FF1 and FF2

In contrast to 3LOC species, secondary homothallic species with flip/flop systems (FF1 and FF2) switch their mating types by inverting a section of chromosome, exchanging *MAT* genes between an expression site and a repression site near a centromere or telomere [7, 13, 14]. The best-characterized species with a flip/flop system is *Ogataea polymorpha*, which we categorize as FF1 (Figures 1C and S1B) because there is one inverted repeat (IR) sequence flanking its *MAT* genes. Recombination between the sequences that form the IR inverts the whole region containing the *MAT* genes in *O. polymorpha*. The FF2 category (Figure 1D) describes species such as *Komagataella phaffii* that also use a flip/flop mechanism to switch mating types but have two IRs, one on each side of their *MAT* genes [13].

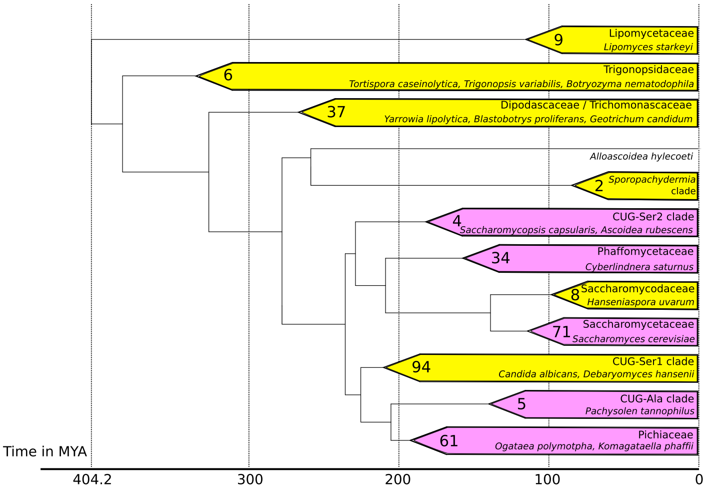

	HET	PHC	PHN	FF1	FF2	3LOC	NOMAT
Lipomycetaceae	1	5	3				
Trigonopsidaceae	6						
Dipodascaceae / Trichomonascaceae	35		2				
Alloascoidea hylecoeti	1						
Sporopachydermia clade	1	1					
CUG-Ser2 clade	2		1	1			
Phaffomycetaceae	24	5		4	1		
Saccharomycodaceae	8						
Saccharomycetaceae	3		2			66	
CUG-Ser1 clade	76	13	3				2
CUG-Ala clade	2		2	1			
Pichiaceae	33	1	3	17	7		
Totals	192	25	16	23	8	66	2

Figure 2. Diversity of Mating Compatibility Systems within Major Clades of Budding Yeasts

For each of the 12 major clades identified by phylogenomic analysis [6], the number of species classified into each of the 7 categories of mating compatibility system is shown in the table on the right. Pink shading indicates clades that include species that switch mating type (FF1, FF2, or 3LOC). For each clade, the number of species is indicated, some representative species are named, and its estimated age is shown by the scale. The tree topology and clade ages are taken from Shen, Opulente, Kominek, Zhou, et al. [6].

See also STAR Methods and Data S1 and S2.

PHC and PHN

We classified species as primary homothallics if they contain both *MATa* and *MATα* genes at non-allelic positions but lack any DNA repeats near these genes. The absence of repeats means that there is no apparent mechanism by which they could switch mating type, in contrast to the secondary homothallics. We defined one group (PHC, primary homothallic contiguous; Figure 1E) as those in which the *MATa* and *MATα* genes are close to each other in the genome (<20 kb apart), as previously seen in species such as *Debaryomyces hansenii* and *Scheffersomyces stipitis*, both of which are considered to be primary homothallics [5, 15–17]. Other genomes in which *MATa* and *MATα* genes are both present on the same contig but far apart (all examples are >90 kb apart), or on different non-allelic contigs, were categorized as PHN (primary homothallic non-contiguous; Figure 1F).

NOMAT

Two of the 332 genomes contained no identifiable *MAT* genes and were classified as NOMAT (Figure 1G; STAR Methods)—*Lodderomyces elongisporus* [18, 19] and *Candida sojae* [20]. The molecular mechanisms that these species use to control cell type and mating are completely unknown.

A Single Origin of the Three-Locus System of Mating-type Switching in Budding Yeasts

Phylogenomic analysis has grouped the 332 budding yeast species into 12 major clades [6], and most clades include species that differ in their mating compatibility systems (Figure 2; Data S1). Approximately half the species in the dataset are heterothallic (192 of 332), and most clades include both heterothallic and homothallic species (Figure 2).

The 3LOC system of mating-type switching is present in almost all the studied species of the family Saccharomycetaceae (66 of 71 species) but occurs nowhere else in the phylogenetic tree of budding yeasts. It is therefore inferred to have originated during the early evolution of Saccharomycetaceae, prior to the deepest divergence within this family, which is the split between the *Kluyveromyces* lineage and the *Saccharomyces* lineage. The five Saccharomycetaceae species that do not have a 3LOC system are *Lachancea kluyveri*, which is HET and has lost the *HML* and *HMR* loci [21, 22], and four species of *Kazachstania*. The four *Kazachstania* species are not monophyletic and represent one transition from the 3LOC system to HET and two separate transitions from 3LOC to primary homothallism (PHN) (Data S1). The best characterized of these PHN *Kazachstania* species is *K. africana*, in which a chromosomal breakage at the ancestral *MAT* locus, together with the loss of *HML* and *HMR*, created a species with non-allelic *MATa* and *MATα* loci on two different chromosomes [23]. These four *Kazachstania* species have all lost the *HO* endonuclease gene, which indicates that they do not switch mating types, whereas the 13 other sequenced *Kazachstania* species retain *HO*.

We infer that there was a transition from HET to 3LOC at the base of the family Saccharomycetaceae because the sister clade Saccharomycodaceae contains only HET species, and the closest outgroup clade (Phaffomycetaceae) contains many HET species and appears to have been HET at its base (Figure 2; Data S1). Nevertheless, such a HET → 3LOC transition in a single step is difficult to envisage because it would require numerous changes to the genome, so unseen intermediate steps may have been involved ([13]; see below).

Although we infer a single origin for the three-locus switching system in budding yeasts, a 3LOC system also evolved in parallel in the fission yeast *Schizosaccharomyces pombe*, which is a member of a different subphylum (Taphrinomycotina) and lies

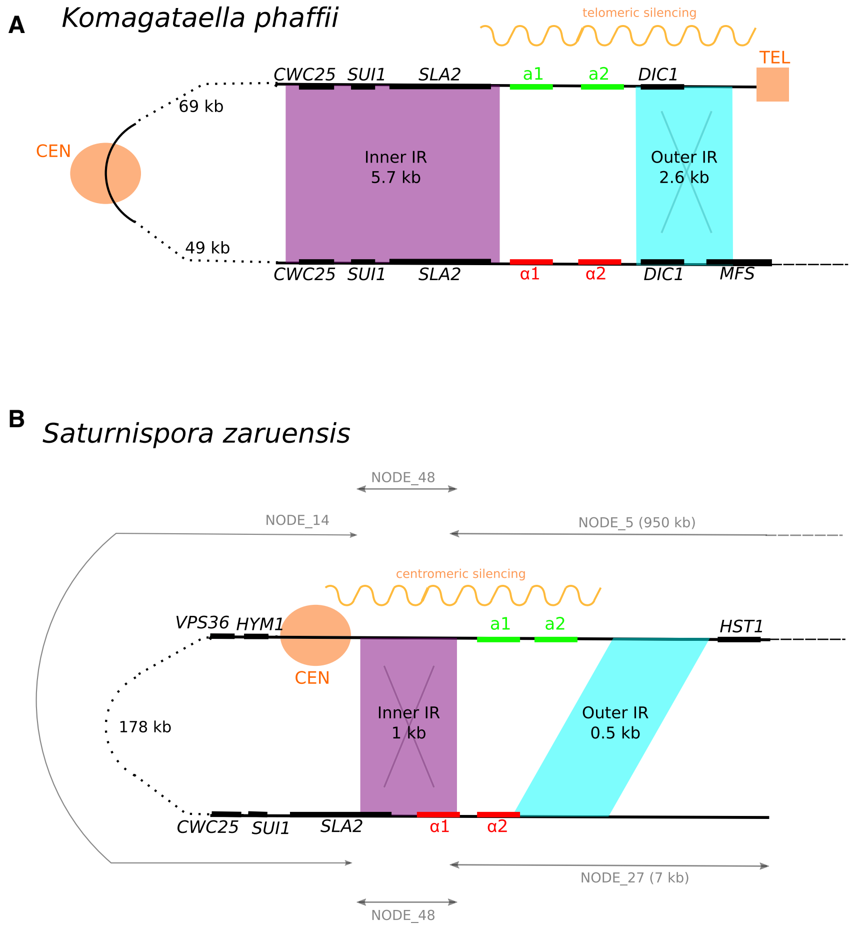

Figure 3. Comparison of *MAT* Locus Organization in Two Species that Use FF2 Systems of Secondary Homothallism

Purple and blue shading indicates inverted repeat (IR) sequences. Centromeres (CEN) and telomeres (TEL) are marked.

(A) Organization in *Komagataella phaffii* [13, 27]. Recombination in the outer IR (blue) is known to move the telomere from beside *MATa* genes to beside *MATα* genes, repressing their transcription.

(B) Organization in *Saturnispora zaruensis*, inferred from the scaffolds (nodes) in its genome assembly. Recombination between the two copies of the inner IR (purple) is inferred to move the centromere from beside *MATa* genes to beside *MATα* genes, repressing their transcription. The total length of the region between the inner IRs is 194 kb.

completely outside the tree in Figure 2. The switching mechanism of *S. pombe* is analogous, not homologous, to the mechanism in *S. cerevisiae* and its mechanistic details are substantially different [5, 24, 25].

At Least 10 Independent Origins of Flip/Flop Mating-type Switching Systems

The most parsimonious interpretation of our data is that the flip/flop mechanism of mating-type switching, in which a section of chromosome becomes inverted, does not have a single evolutionary origin but arose independently 10–11 times in different lineages. There are eight independent clades with FF1 systems and two or three with FF2 systems (Data S1). Most of these flip/flop clades are phylogenetically quite narrow and therefore quite young (nine of the 11 are <100 million years old [6]).

The eight FF1 clades include three that have been reported previously, and five newly discovered ones. The previously known ones are clades containing *Pachysolen tannophilus*, *Ascoidea rubescens*, and *Ogataea polymorpha* [5, 7]. *P. tannophilus* and *A. rubescens* are both singleton FF1 species whose closest relatives are heterothallic (Data S1, S2A, and S2B). *O. polymorpha* lies within a clade of 12 *Ogataea* species that all switch by an FF1 mechanism [13, 14, 26], but the genus *Ogataea* also contains two other clades with newly discovered FF1 systems that we infer to have originated independently of the 12-species *O. polymorpha* FF1 group (Data S1; STAR Methods).

Other newly discovered FF1 systems occur in the genera *Cyberlindnera*, *Starmera*, and *Kregervanrija*, representing three additional independent origins of FF1 (Data S1). In *Cyberlindnera* (Phaffomycetaceae), a clade of three species with FF1 secondary homothallism is nested inside this otherwise heterothallic genus. These three species (*C. saturnus*, *C. mrakii*, and *C. suaveolens*) contain an invertible region of approximately 49 kb spanning 13 genes, with *MATa* genes at one end and *MATα* genes at the other end, flanked by an IR (Data S2C). One set of *MAT* genes, located between *SLA2* and *VPS75*, is orthologous to the single *MAT* locus of *C. jadinii* (HET, diploid). *Starmera quercuum* (Phaffomycetaceae) contains a similar but larger invertible region of 61 kb, flanked by an IR that extends to the ends of the available contig, while in *Kregervanrija* (Pichiaceae), the two studied species both contain only the four canonical *MAT* genes on a 12-kb invertible region flanked by IRs (Data S2C).

The two clades with unambiguous FF2 systems are species of *Komagataella* and *Saturnispora*, which are both in family Pichiaceae but only distantly related to each other. The *Komagataella* FF2 system has been characterized in detail in *K. phaffii* (Figure 3A) and is shared by two other studied species of *Komagataella* (Data S1). In these species, the repressed *MAT* locus is located near a telomere [13, 28]. The region that inverts is 138 kb long and includes about 72 genes and a centromere [27].

The *Saturnispora* FF2 system, illustrated by *S. zaruensis* (Figure 3B), is newly discovered. It is present in a clade of four species within this genus, whereas three outgroup *Saturnispora* species are heterothallic (Data S1). The *S. zaruensis MATa* and *MATα* genes are almost 200 kb apart and flanked by two IRs, one on each side of the *MAT* genes (Figure 3B). We infer that there is a centromere located asymmetrically inside the 194-kb invertible region, close to one end (STAR Methods). The *S. zaruensis* system appears to use centromeric repression of transcription, in contrast to the telomeric repression seen in *K. phaffii*. Recombination between the inner IR sequences

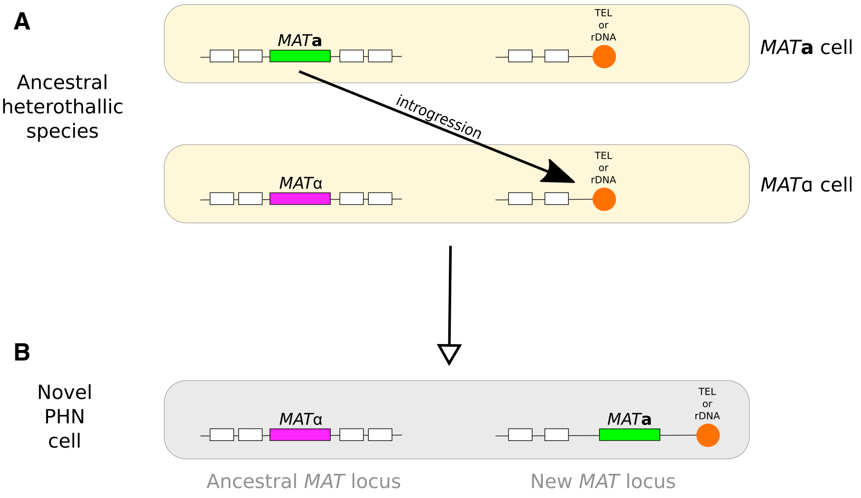

Figure 4. Proposed Mechanism of Transition from Heterothallism to Non-contiguous Primary Homothallism (PHN) by Introgression

White boxes at each locus represent genes syntenic among all three cells, directly below one another.

(A) Two haploid cells of the ancestral heterothallic species. A DNA introgression event transfers or copies the single *MAT* locus of one cell into another cell of the opposite mating type, possibly via a cycle of mating, genomic rearrangement and sporulation. The introgressed DNA is integrated into the recipient genome, either close to a telomere (as observed in the genus *Nadsonia*) or close to an rDNA array (as observed in the genus *Peterozyma*), marked in orange.

(B) The resulting haploid cell has two *MAT* loci in a PHN arrangement.

(purple in Figure 3B) would cause the centromere to move from close to the *MATa* genes to close to the *MATα* genes, potentially switching repression of transcription from one set of genes to the other. The newly discovered FF2 system in *S. zaruensis* and the previously studied one in *K. phaffii* are both characterized by a relatively long distance between the two *MAT* loci, unlike most of the FF1 systems. We have previously postulated that the function of the second repeat is to restore colinearity of the chromosome in diploid cells and enable meiotic recombination. The second set of repeats is known to be functional in *K. phaffii*, as evidenced by different orientations of the flip-flopping regions among natural isolates of *K. phaffii* [13].

We provisionally classified *Wickerhamomyces canadensis* (Phaffomycetaceae) as a third independent FF2 system (Data S2D). Its closest relatives are heterothallic. In *W. canadensis*, a *MATa* locus is present in the middle of a large (612 kb) scaffold, and a *MATα* locus is present on a small (7 kb) contig that is probably subtelomeric (STAR Methods). The *MAT* genes are flanked by two repeat sequences: a 2-kb repeat made from the 3′ end of *SLA2*, and a 0.2-kb repeat that includes the 5′ end of *DIC1*. This organization resembles the organization of the *Komagataella phaffii MAT* loci, but its assignment as FF2 is not certain because we do not know whether the two *MAT* loci are on the same chromosome, or the relative orientations of the repeats.

Interestingly, all of the 11 clades with secondary homothallic systems are inferred to have evolved from heterothallic ancestors (Figure 1). We did not detect any transitions between different types of secondary homothallism, nor any transitions from primary to secondary homothallism. The existence of multiple independent FF2 clades naturally suggests a series of transitions that could lead to the emergence of a 3LOC system: HET → FF1 → FF2 → 3LOC, first postulated by Hanson et al. [13]. In it, the transition from FF1 to FF2 could be caused by a growing distance between the two *MAT* loci, leading to selection for a second IR to restore colinearity of the chromosome. Subsequently, duplication of one of the two IR-flanked *MAT* loci could create a species with three *MAT* loci in a genomic arrangement very similar to that of a functional 3LOC system. Nevertheless, the intermediate transitions required by this scenario are not observed in our data.

Conversion of Heterothallics to Primary Homothallics by Introgression of *MAT* Genes at Telomeric and rDNA Sites

We identified a clear case of a heterothallic ancestor producing a primary homothallic (PHN) descendant, in the genus *Nadsonia* (Figure 4; Data S2E). Two species in this genus, *N. fulvescens* var. *fulvescens* [6] and *N. starkeyi-henricii* [29], have a single, orthologous, *MAT* locus (with *MATα* and *MATa* genotypes, respectively) and so are classified as heterothallic. A third species, *N. fulvescens* var. *elongata* [7], has *MATα* genes at this locus but also has *MATa* genes on another scaffold, at a site that is close to a telomere (Data S2E). Comparison between the two *N. fulvescens* varieties indicates that a few kilobases of DNA, including the *MATa* genes, have been gained by var. *elongata* at this telomere, converting it from a heterothallic species to a primary homothallic (PHN) species. Consistent with these genome-based designations of mating compatibility systems, *N. fulvescens* var. *elongata* is homothallic (sporulation occurs following conjugation between a cell and its bud), whereas the other two species have no known sexual cycle and do not form spores [30].

Similar to the situation we described for *Nadsonia fulvescens* var. *elongata*, many of the other species we classified as PHN also contain one old *MAT* locus at a site syntenic with the *MAT* loci of closely related HET species, and a new second *MAT* locus with the opposite *MAT* allele, at a location that is close to a telomere. The PHN species in this category are *Blastobotrys proliferans* (Trichomonascaceae), *Kuraishia molischiana* (Pichiaceae), two *Yamadazyma* species (CUG-Ser1 clade), *Wickerhamia fluorescens* (CUG-Ser1 clade), *Saccharomycopsis capsularis* (CUG-Ser2 clade), two *Lipomyces* species (*L. oligophaga* and *L. suomiensis*; Lipomycetaceae), *Kazachstania rosinii* (Saccharomycetaceae), and *Nadsonia fulvescens* var. *elongata.* In four of these species, the new subtelomeric *MAT* locus is beside a pseudogene of *SLA2*. Thus, among the 12 transitions to PHN in our dataset (Figure 1), eight of them involved the gain of DNA containing the opposite *MAT* allele, at a site near a telomere. We hypothesize that these events are the result of DNA introgression between strains that were initially heterothallic. A related situation occurs in two *Peterozyma* species (CUG-Ala clade) that became PHN by gaining a second *MAT* locus at a site beside the rDNA array (Figure 4; Data S2B).

Genomic regions close to rDNA, telomeres, and centromeres can form heterochromatin that represses transcription, and it is striking that almost every example of PHN that we detected involves gain of a new *MAT* locus either near a telomere or beside the rDNA. Furthermore, the genomic rearrangement that converted *Kazachstania africana* from 3LOC to PHN left one of its two *MAT* loci in a subtelomeric region [23]. In fact, only two of the 12 PHN clades in our dataset have a second *MAT* locus that is not telomere or rDNA associated, and in both of these clades (*Lipomyces japonicus* and a pair of *Ambrosiozyma* species) there is a large, possibly heterochromatic, region of non-coding DNA beside the second *MAT* locus (STAR Methods).

The pattern of PHN species emerging by gaining a second *MAT* locus in a heterochromatic region of the genome (Figure 4) may indicate that these second *MAT* loci are silenced at some stage in the life cycle of the organisms that contain them. Cells of primary homothallic species contain both *MATa* and *MAT*α genes, but the molecular details of how these genes control mating and sporulation are not understood (how does each cell know whether it should mate or sporulate?). We agree with previous speculations that primary homothallic species may use epigenetic or other regulatory mechanisms to ensure that each haploid cell expresses only *MATa* or *MAT*α genes, even though both types of gene are present in the genome [4, 31]. If correct, this model would mean that true primary homothallism does not exist, i.e., that, even in primary homothallic species, mating is between two cells that are transcriptionally *MATa* and *MAT*α. Alternatively, the PHN systems could potentially be examples of tetrapolar mating systems [3], but so little is known about the genetics of the species that contain them or the functions of their *MAT* genes, that the consequences of having separate, unlinked, *MATa* and *MAT*α loci in these yeasts cannot be predicted. A third possibility is that the bias toward subtelomeric and rDNA sites may simply reflect the receptiveness of these sites toward introgressed DNA [6, 32].

Evolutionary Transitions toward Homothallism

In the entire dataset of 332 yeast species, we see 31 transitions from heterothallism to homothallism, but only three instances of transition in the opposite direction (Figure 1). The asymmetry between these numbers is striking, particularly considering the likely molecular mechanisms of transition. Naively, we would expect transitions from heterothallism to homothallism to be difficult, because they require a complex series of steps: introgression of *MAT* genes to create a primary homothallic species or relocations of *MAT* genes to silenced positions near the centromere or telomere, as well as DNA duplications to make repeats, to create a secondary homothallic species. In contrast, a homothallic cell can become heterothallic very easily, by just deleting some DNA [33].

The 31 transitions to homothallism comprise 19 to primary homothallism and 12 to secondary homothallism (Figure 1). The three transitions away from homothallism consist of two 3LOC → HET transitions that occurred in *Lachancea kluyveri* [22] and a pair of *Kazachstania* species, and a PHC → HET transition in *Lipomyces doorenjongii* (Data S1 and S2F; STAR Methods).

Our analysis shows clearly that evolutionary pressure has repeatedly led to the emergence of homothallic descendants from heterothallic ancestors. The fact that homothallism was gained 31 times and lost only 3 times suggests that it has overwhelming evolutionary benefits, as has been postulated in the past [3, 13, 34, 35]. We have suggested that the evolutionary benefit of homothallism is that it gives a yeast species the ability to form new spores during the first few cell generations after spore germination, thereby removing the death penalty that too-early germination otherwise entails if the environment is inadequate [13, 36]. Alternatively, homothallism may have been favored by selection because it enables reproductive assurance [35, 37], diploidization and DNA repair [9], or genome renewal [38, 39]. The large number of independent origins of homothallism, in both its primary and secondary forms, points to DNA rearrangements of the *MAT* locus being an effective, albeit radical, way of increasing the fitness of a budding yeast species.

STAR★METHODS

Detailed methods are provided in the online version of this paper and include the following:

- KEY RESOURCES TABLE
- LEAD CONTACT AND MATERIALS AVAILABILITY
- EXPERIMENTAL MODEL AND SUBJECT DETAILS
- METHOD DETAILS
  - Saccharomycetaceae
  - Saccharomycodaceae
  - Phaffomycetaceae
  - CUG-Ser2 clade
  - CUG-Ser1 clade
  - Pichiacae
  - CUG-Ala clade
  - Sporopachydermia
  - Dipodascaceae/Trichomonascaceae
  - Trigonopsidaceae
  - Lipomycetacae
- QUANTIFICATION AND STATISTICAL ANALYSIS
- DATA AND CODE AVAILABILITY

SUPPLEMENTAL INFORMATION

Supplemental Information can be found online at https://doi.org/10.1016/j.cub.2019.06.056.

ACKNOWLEDGMENTS

This work was supported by the Wellcome Trust PhD programme in Computational Infection Biology at University College Dublin (109165/Z/15/Z), Science Foundation Ireland (13/IA/1910), and the European Research Council (789341). It is based on work supported by the National Science Foundation under grant nos. DEB-1442113 and DEB-1442148 and funded in part by the DOE Great Lakes Bioenergy Research Center (DOE Office of Science BER DE-SC0018409). C.T.H. is a Pew Scholar in the Biomedical Sciences and Vilas Faculty Early Career Investigator, supported by the Pew Charitable Trusts and Vilas Trust Estate, respectively. A.R. acknowledges a Guggenheim Fellowship. We thank Kevin Byrne and other members of the Wolfe lab for assistance and comments on the manuscript.

AUTHOR CONTRIBUTIONS

Conceptualization, K.H.W., T.K., and C.T.H.; Methodology, T.K. and K.H.W.; Software, T.K.; Investigation, T.K., K.H.W., J.K., D.A.O., X.-X.S., and X.Z.;

Writing – Original Draft, K.H.W. and T.K.; Writing – Review & Editing, K.H.W., T.K., C.T.H., A.R., and J.K.; Funding Acquisition, K.H.W., T.K., C.T.H., and A.R.; Resources, K.H.W., C.T.H., J.K., D.A.O., A.R., X.-X.S., and X.Z.; Supervision, K.H.W. and C.T.H.; Data Curation, T.K. and J.K.

DECLARATION OF INTERESTS

The authors declare no competing interests.

Received: April 8, 2019
Revised: June 8, 2019
Accepted: June 20, 2019
Published: July 25, 2019

REFERENCES

1. C.P. Kurtzman, J.W. Fell, and T. Boekhout, eds. (2011). The Yeasts, a Taxonomic Study (Elsevier).
2. Wilson, A.M., Wilken, P.M., van der Nest, M.A., Steenkamp, E.T., Wingfield, M.J., and Wingfield, B.D. (2015). Homothallism: an umbrella term for describing diverse sexual behaviours. IMA Fungus *6*, 207–214.
3. Lin, X., and Heitman, J. (2007). Mechanisms of homothallism in fungi and transitions between heterothallism and homothallism. In Sex in Fungi, J. Heitman, J.W. Kronstad, J.W. Taylor, and L.A. Casselton, eds. (ASM Press), pp. 35–57.
4. Muller, H., Hennequin, C., Dujon, B., and Fairhead, C. (2007). Ascomycetes: the *Candida MAT* locus: comparing *MAT* in the genomes of hemiascomycetous yeasts. In Sex in Fungi, J. Heitman, J.W. Kronstad, J.W. Taylor, and L.A. Casselton, eds. (ASM Press), pp. 247–263.
5. Hanson, S.J., and Wolfe, K.H. (2017). An evolutionary perspective on yeast mating-type switching. Genetics *206*, 9–32.
6. Shen, X.X., Opulente, D.A., Kominek, J., Zhou, X., Steenwyk, J.L., Buh, K.V., Haase, M.A.B., Wisecaver, J.H., Wang, M., Doering, D.T., et al. (2018). Tempo and mode of genome evolution in the budding yeast subphylum. Cell *175*, 1533–1545.
7. Riley, R., Haridas, S., Wolfe, K.H., Lopes, M.R., Hittinger, C.T., Göker, M., Salamov, A.A., Wisecaver, J.H., Long, T.M., Calvey, C.H., et al. (2016). Comparative genomics of biotechnologically important yeasts. Proc. Natl. Acad. Sci. USA *113*, 9882–9887.
8. Inderbitzin, P., and Turgeon, B.G. (2015). Pondering mating: *Pneumocystis jirovecii*, the human lung pathogen, selfs without mating type switching, in contrast to Its close relative *Schizosaccharomyces pombe*. MBio *6*. Published online May 5, 2015. https://doi.org/10.1128/mBio.00583-15.
9. Herskowitz, I. (1988). Life cycle of the budding yeast *Saccharomyces cerevisiae*. Microbiol. Rev. *52*, 536–553.
10. Haber, J.E. (2012). Mating-type genes and *MAT* switching in *Saccharomyces cerevisiae*. Genetics *191*, 33–64.
11. Barsoum, E., Martinez, P., and Aström, S.U. (2010). Alpha3, a transposable element that promotes host sexual reproduction. Genes Dev. *24*, 33–44.
12. Rajaei, N., Chiruvella, K.K., Lin, F., and Aström, S.U. (2014). Domesticated transposase Kat1 and its fossil imprints induce sexual differentiation in yeast. Proc. Natl. Acad. Sci. USA *111*, 15491–15496.
13. Hanson, S.J., Byrne, K.P., and Wolfe, K.H. (2014). Mating-type switching by chromosomal inversion in methylotrophic yeasts suggests an origin for the three-locus *Saccharomyces cerevisiae* system. Proc. Natl. Acad. Sci. USA *111*, E4851–E4858.
14. Maekawa, H., and Kaneko, Y. (2014). Inversion of the chromosomal region between two mating type loci switches the mating type in *Hansenula polymorpha*. PLoS Genet. *10*, e1004796.
15. Suzuki, M., Prasad, G.S., and Kurtzman, C.P. (2011). *Debaryomyces* Lodder & Kreger-van Rij (1952). In The Yeasts, A Taxonomic Study, *Volume 2*, C.P. Kurtzman, J.W. Fell, and T. Boekhout, eds. (Elsevier), pp. 361–372.
16. Kurtzman, C.P. (2011). *Scheffersomyces* Kurtzman & M. Suzuki (2010). In The Yeasts, A Taxonomic Study, *Volume 2*, C.P. Kurtzman, J.W. Fell, and T. Boekhout, eds. (Elsevier), pp. 773–777.
17. Wolfe, K.H., and Butler, G. (2017). Evolution of mating in the Saccharomycotina. Annu. Rev. Microbiol. *71*, 197–214.
18. Butler, G., Rasmussen, M.D., Lin, M.F., Santos, M.A., Sakthikumar, S., Munro, C.A., Rheinbay, E., Grabherr, M., Forche, A., Reedy, J.L., et al. (2009). Evolution of pathogenicity and sexual reproduction in eight *Candida* genomes. Nature *459*, 657–662.
19. Döğen, A., Metin, B., Ilkit, M., de Hoog, G.S., and Heitman, J. (2017). MTL genotypes, phenotypic switching, and susceptibility profiles of *Candida parapsilosis* species group compared to *Lodderomyces elongisporus*. PLoS ONE *12*, e0182653.
20. Borelli, G., José, J., Teixeira, P.J., Dos Santos, L.V., and Pereira, G.A. (2016). *De novo* assembly of *Candida sojae* and *Candida boidinii* genomes, unexplored xylose-consuming yeasts with potential for renewable biochemical production. Genome Announc. *4*. Published online January 14, 2016. https://doi.org/10.1128/genomeA.01551-15.
21. Souciet, J.L., Dujon, B., Gaillardin, C., Johnston, M., Baret, P.V., Cliften, P., Sherman, D.J., Weissenbach, J., Westhof, E., Wincker, P., et al.; Génolevures Consortium (2009). Comparative genomics of protoploid Saccharomycetaceae. Genome Res. *19*, 1696–1709.
22. Vakirlis, N., Sarilar, V., Drillon, G., Fleiss, A., Agier, N., Meyniel, J.P., Blanpain, L., Carbone, A., Devillers, H., Dubois, K., et al. (2016). Reconstruction of ancestral chromosome architecture and gene repertoire reveals principles of genome evolution in a model yeast genus. Genome Res. *26*, 918–932.
23. Wolfe, K.H., Armisén, D., Proux-Wera, E., ÓhÉigeartaigh, S.S., Azam, H., Gordon, J.L., and Byrne, K.P. (2015). Clade- and species-specific features of genome evolution in the Saccharomycetaceae. FEMS Yeast Res. *15*, fov035.
24. Nielsen, O., and Egel, R. (2007). The *mat* genes of *Schizosaccharomyces pombe*: expression, homothallic switch, and silencing. In Sex in Fungi, J. Heitman, J.W. Kronstad, J.W. Taylor, and L.A. Casselton, eds. (ASM Press), pp. 143–157.
25. Klar, A.J., Ishikawa, K., and Moore, S. (2014). A unique DNA recombination mechanism of the mating/cell-type switching of fission yeasts: a review. Microbiology Spectrum *2*, MDNA3-0003-2014.
26. Yoko-O, T., Komatsuzaki, A., Yoshihara, E., Umemura, M., and Chiba, Y. (2019). Mating type switching, formation of diploids, and sporulation in the methylotrophic yeast *Ogataea minuta*. J. Biosci. Bioeng. *127*, 1–7.
27. Coughlan, A.Y., Hanson, S.J., Byrne, K.P., and Wolfe, K.H. (2016). Centromeres of the yeast *Komagataella phaffii (Pichia pastoris)* have a simple inverted-repeat structure. Genome Biol. Evol. *8*, 2482–2492.
28. Heistinger, L., Gasser, B., and Mattanovich, D. (2017). Creation of stable heterothallic strains of *Komagataella phaffii* enables dissection of mating gene regulation. Mol. Cell. Biol. *38*, e00398.
29. O'Boyle, S., Bergin, S.A., Hussey, E.E., McLaughlin, A.D., Riddell, L.R., Byrne, K.P., Wolfe, K.H., O'Brien, C.E., and Butler, G. (2018). Draft genome sequence of the yeast *Nadsonia starkeyi-henricii* UCD142, isolated from forest soil in Ireland. Genome Announc. Published online June 21, 2018. https://doi.org/10.1128/genomeA.00549-18.
30. Smith, M.T. (2011). *Nadsonia* Sydow (1912). In The Yeasts, a Taxonomic Study, C.P. Kurtzman, J.W. Fell, and T. Boekhout, eds. (Elsevier), pp. 629–632.
31. Coppin, E., Debuchy, R., Arnaise, S., and Picard, M. (1997). Mating types and sexual development in filamentous ascomycetes. Microbiol. Mol. Biol. Rev. *61*, 411–428.
32. Peter, J., De Chiara, M., Friedrich, A., Yue, J.X., Pflieger, D., Bergström, A., Sigwalt, A., Barre, B., Freel, K., Llored, A., et al. (2018). Genome evolution across 1,011 *Saccharomyces cerevisiae* isolates. Nature *556*, 339–344.
33. Yun, S.H., Berbee, M.L., Yoder, O.C., and Turgeon, B.G. (1999). Evolution of the fungal self-fertile reproductive life style from self-sterile ancestors. Proc. Natl. Acad. Sci. USA *96*, 5592–5597.

34. Knop, M. (2006). Evolution of the hemiascomycete yeasts: on life styles and the importance of inbreeding. BioEssays *28*, 696–708.
35. Nieuwenhuis, B.P., and Immler, S. (2016). The evolution of mating-type switching for reproductive assurance. BioEssays *38*, 1141–1149.
36. Gordon, J.L., Armisén, D., Proux-Wéra, E., ÓhÉigeartaigh, S.S., Byrne, K.P., and Wolfe, K.H. (2011). Evolutionary erosion of yeast sex chromosomes by mating-type switching accidents. Proc. Natl. Acad. Sci. USA *108*, 20024–20029.
37. Nieuwenhuis, B.P.S., Tusso, S., Bjerling, P., Stångberg, J., Wolf, J.B.W., and Immler, S. (2018). Repeated evolution of self-compatibility for reproductive assurance. Nat. Commun. *9*, 1639.
38. Mortimer, R.K. (2000). Evolution and variation of the yeast (*Saccharomyces*) genome. Genome Res. *10*, 403–409.
39. Magwene, P.M. (2014). Revisiting Mortimer's genome renewal hypothesis: heterozygosity, homothallism, and the potential for adaptation in yeast. In Ecological Genomics: Ecology and the Evolution of Genes and Genomes. Advances in Experimental Medicine and Biology 781, C.R. Landry, and N. Aubin-Horth, eds. (Springer), pp. 37–48.
40. Altschul, S.F., Madden, T.L., Schäffer, A.A., Zhang, J., Zhang, Z., Miller, W., and Lipman, D.J. (1997). Gapped BLAST and PSI-BLAST: a new generation of protein database search programs. Nucleic Acids Res. *25*, 3389–3402.
41. Gouy, M., Guindon, S., and Gascuel, O. (2010). SeaView version 4: a multiplatform graphical user interface for sequence alignment and phylogenetic tree building. Mol. Biol. Evol. *27*, 221–224.
42. Bankevich, A., Nurk, S., Antipov, D., Gurevich, A.A., Dvorkin, M., Kulikov, A.S., Lesin, V.M., Nikolenko, S.I., Pham, S., Prjibelski, A.D., et al. (2012). SPAdes: a new genome assembly algorithm and its applications to single-cell sequencing. J. Comput. Biol. *19*, 455–477.
43. Weisenfeld, N.I., Yin, S., Sharpe, T., Lau, B., Hegarty, R., Holmes, L., Sogoloff, B., Tabbaa, D., Williams, L., Russ, C., et al. (2014). Comprehensive variation discovery in single human genomes. Nat. Genet. *46*, 1350–1355.
44. Wickerham, L.J. (1958). Sexual agglutination of heterothallic yeasts in diverse taxonomic areas. Science *128*, 1504–1505.
45. Kellis, M., Patterson, N., Endrizzi, M., Birren, B., and Lander, E.S. (2003). Sequencing and comparison of yeast species to identify genes and regulatory elements. Nature *423*, 241–254.
46. Scannell, D.R., Zill, O.A., Rokas, A., Payen, C., Dunham, M.J., Eisen, M.B., Rine, J., Johnston, M., and Hittinger, C.T. (2011). The awesome power of yeast evolutionary genetics: New genome sequences and strain resources for the *Saccharomyces sensu stricto* genus. G3 (Bethesda) *1*, 11–25.
47. Liti, G., Nguyen Ba, A.N., Blythe, M., Müller, C.A., Bergström, A., Cubillos, F.A., Dafhnis-Calas, F., Khoshraftar, S., Malla, S., Mehta, N., et al. (2013). High quality de novo sequencing and assembly of the *Saccharomyces arboricolus* genome. BMC Genomics *14*, 69.
48. Gabaldón, T., Martin, T., Marcet-Houben, M., Durrens, P., Bolotin-Fukuhara, M., Lespinet, O., Arnaise, S., Boisnard, S., Aguileta, G., Atanasova, R., et al. (2013). Comparative genomics of emerging pathogens in the *Candida glabrata* clade. BMC Genomics *14*, 623.
49. Galeote, V., Bigey, F., Devillers, H., Ortiz-Merino, R.A., Dequin, S., Wolfe, K.H., and Neuvéglise, C. (2018). Genome Sequence of *Torulaspora microellipsoides* CLIB 830^{T}. Genome Announc. *6*, e00615–e00618.
50. Galeote, V., Bigey, F., Devillers, H., Neuvéglise, C., and Dequin, S. (2013). Genome sequence of the food spoilage yeast *Zygosaccharomyces bailii* CLIB 213T. Genome Announc. *1*, e00606–e00613.
51. Ortiz-Merino, R.A., Kuanyshev, N., Braun-Galleani, S., Byrne, K.P., Porro, D., Branduardi, P., and Wolfe, K.H. (2017). Evolutionary restoration of fertility in an interspecies hybrid yeast, by whole-genome duplication after a failed mating-type switch. PLoS Biol. *15*, e2002128.
52. Wendland, J., and Walther, A. (2011). Genome evolution in the eremothecium clade of the Saccharomyces complex revealed by comparative genomics. G3 (Bethesda) *1*, 539–548.
53. Freel, K.C., Friedrich, A., Sarilar, V., Devillers, H., Neuvéglise, C., and Schacherer, J. (2016). Whole-genome sequencing and intraspecific analysis of the yeast species *Lachancea quebecensis*. Genome Biol. Evol. *8*, 733–741.
54. Sarilar, V., Devillers, H., Freel, K.C., Schacherer, J., and Neuvéglise, C. (2015). Draft genome sequence of *Lachancea lanzarotensis* CBS 12615T, an ascomycetous yeast isolated from grapes. Genome Announc. *3*. Published online April 16, 2015. https://doi.org/10.1128/genomeA.00292-15.
55. Hull, C.M., and Johnson, A.D. (1999). Identification of a mating type-like locus in the asexual pathogenic yeast *Candida albicans*. Science *285*, 1271–1275.
56. Wohlbach, D.J., Kuo, A., Sato, T.K., Potts, K.M., Salamov, A.A., Labutti, K.M., Sun, H., Clum, A., Pangilinan, J.L., Lindquist, E.A., et al. (2011). Comparative genomics of xylose-fermenting fungi for enhanced biofuel production. Proc. Natl. Acad. Sci. USA *108*, 13212–13217.
57. Douglass, A.P., Offei, B., Braun-Galleani, S., Coughlan, A.Y., Martos, A.A.R., Ortiz-Merino, R.A., Byrne, K.P., and Wolfe, K.H. (2018). Population genomics shows no distinction between pathogenic *Candida krusei* and environmental *Pichia kudriavzevii*: one species, four names. PLoS Pathog. *14*, e1007138.
58. Gonçalves, C., Wisecaver, J.H., Kominek, J., Oom, M.S., Leandro, M.J., Shen, X.X., Opulente, D.A., Zhou, X., Peris, D., Kurtzman, C.P., et al. (2018). Evidence for loss and reacquisition of alcoholic fermentation in a fructophilic yeast lineage. eLife *7*. Published online April 12, 2018. https://doi.org/10.7554/eLife.33034.
59. Kunze, G., Gaillardin, C., Czernicka, M., Durrens, P., Martin, T., Böer, E., Gabaldón, T., Cruz, J.A., Talla, E., Marck, C., et al. (2014). The complete genome of *Blastobotrys (Arxula) adeninivorans* LS3 - a yeast of biotechnological interest. Biotechnol. Biofuels *7*, 66.
60. Cohn, M., McEachern, M.J., and Blackburn, E.H. (1998). Telomeric sequence diversity within the genus *Saccharomyces*. Curr. Genet. *33*, 83–91.

STAR★METHODS

KEY RESOURCES TABLE

REAGENT or RESOURCE	SOURCE	IDENTIFIER
Deposited Data		
The genomes used in the study	DDBJ/ENA/GenBank	Table S1 in [6]
Software and Algorithms		
BLAST	[40]	RRID: SCR_004870
Seaview	[41]	RRID: SCR_015059

LEAD CONTACT AND MATERIALS AVAILABILITY

Further information and requests for resources should be directed to and will be fulfilled by the Lead Contact, Kenneth Wolfe (kenneth.wolfe@ucd.ie). This study did not generate new unique reagents.

EXPERIMENTAL MODEL AND SUBJECT DETAILS

We analyzed the same dataset of 332 budding yeast genome sequences as in Shen, Opulente, Kominek, Zhou, et al. [6], which combined previously published genomes of 112 species with 220 newly sequenced genomes (Data S1).

METHOD DETAILS

For each species we examined only one reference genome sequence assembly [6], which means that for heterothallic (HET) species we may have detected only one of the two possible *MAT* alleles, depending on the nature of the assembly. Because of the high sequence diversity of *MAT* genes, we used a strategy of multiple automated TBLASTN searches [40] against each genome sequence, with diverse sets of *MAT* protein sequences (**a**1, **a**2, α1, α2) as queries, retaining even very weak hits (TBLASTN $E < 10$). Genomic regions that were hit by more than one different *MAT* protein were then examined manually. As additional *MAT* genes were found, they were added to the query dataset. Automated searches were also used to annotate genes commonly located near *MAT* loci, such as *SLA2* and *DIC1*, and to label repeated DNA sequences in the vicinity of *MAT*-like loci that could form parts of IR, X or Z regions. These repeats often contain duplicated parts of *MAT* genes, so care was taken to distinguish between intact genes and gene fragments. When annotating genomes, we interpreted genomic regions containing an intact **a**1 and/or **a**2 gene to be *MAT***a** loci, and regions containing an intact α1 and/or α2 gene to be *MAT*α loci (i.e., we did not require both of the **a** genes or both of the α genes to be present). All cases of inferred absence of one but not both of the genes in a pair (e.g., if α1 is present but α2 is absent, etc), as well as all NOMAT cases, were verified by manual BLAST searches.

For some species, the structure of *MAT*-like regions was inferred from multiple contigs in the genome assembly. In genomes assembled using SPAdes [42], any repeat regions that exist as two highly similar copies in the real genome (such as IRs, X and Z regions) usually co-assemble into single contigs whose coverage is approximately twice the average coverage. These contigs often have short sequence overlaps with the contigs that flank them on each side; the overlaps are the same length as the longest *k*-mer used by SPAdes. By detecting these overlaps and comparing contig coverage, we were able to infer the overall organization of the *MAT*-like regions manually for most species.

We classified species into the 3LOC, FF1 and FF2 categories of secondary homothallism based on the presence of both *MAT***a** and *MAT*α genes in the genome at non-allelic positions, the presence of repeat sequences (IRs or X/Z regions), the numbers of copies of *MAT*-like and repeat sequences, and the presence of truncated genes such as often occur in IRs and X/Z regions [36]. We classified genomes as primary homothallic if they contain both *MAT***a** and *MAT*α genes at non-allelic positions that are not near repeated sequences, and classified them as PHC or PHN depending on whether the distance between *MAT***a** and *MAT*α genes was less than 20 kb. All the examples of PHN that we report have their two *MAT* loci on different non-allelic contigs, or > 90 kb apart on the same contig.

Transitions between mating compatibility systems were inferred manually using parsimony, by reference to the genome structures at *MAT*-like loci and the phylogenomic tree [6] (Data S1). We considered parsimony to be the the most appropriate method, because the number of transitions is low relative to the number of species, and because we have no way of weighting *a priori* the probability of transitions between different systems.

Most (118) of the 220 genomes that were newly sequenced for the Y1000+ Project [6] were assembled using the SPAdes assembler [42]. We noticed that two genomes (*Cyberlindnera saturnus* and *Starmera quercuum*) that were assembled using a different assembler, DISCOVAR [43], each contain a very large inverted repeat (>100 kb) flanking the *MAT* genes (Data S2C). In each case

the two copies of the repeat differ by only 1 nucleotide, and extend to the ends of the assembled contig. The Illumina sequencing protocol used by Shen, Opulente, Kominek, Zhou, et al. [6] does not have power to resolve such large near-identical repeats, so we concluded that these structures are artifacts that occur when FF1 genomes are assembled using DISCOVAR. They do not occur with the SPAdes assembler.

In the remainder of this section we describe the genomic data supporting our inferences of mating compatibility systems, sorted by clade. For species in which we found only *MAT***a** genes or only *MAT*α genes, and no other evidence is available, we inferred that the species is heterothallic (HET) and do not describe it. In all other cases, the rationale for classifying each species into its category is provided below.

Saccharomycetaceae

71 species: genera *Saccharomyces*, *Nakaseomyces, Kazachstania, Naumovozyma, Tetrapisispora, Vanderwaltozyma, Yueomyces, Torulaspora, Zygotorulaspora, Zygosaccharomyces*, *Lachancea, Eremothecium, Ashbya, Kluyveromyces,* and species *Candida nivariensis, Candida bracarensis, Candida glabrata* and *Candida castellii.*

The vast majority of species in family Saccharomycetaceae are capable of mating type switching using a system homologous to that of *Saccharomyces cerevisiae* and are classified as 3LOC. We identified three exceptions to this rule in the genus *Kazachstania*, and one exception in the genus *Lachancea*:

Whereas most species in the genus *Kazachstania* have a 3LOC organization, four of them do not. These four species fall into three separate clades within the genus: *K. africana*, *K. rosinii*, and the pair *K. yakushimaensis*/*K. transvaalensis*. The genome assemblies of each of these four species contain both *MAT***a** and *MAT*α genes. None of the 4 has an *HO* endonuclease gene; these are the only known post-WGD species that do not have an *HO* gene. Moreover, in these species the Yα region does not end at the HO cleavage site in the *MAT*α1 gene, as it does in HO-containing species [36]. Together, these observations indicate that these four *Kazachstania* species have lost the ability to switch mating type. The four are discussed below.

Kazachstania africana has sustained a genomic rearrangement that we have described in detail elsewhere [23], which was probably the result of chromosome breakage at the ancestral *MAT* locus. We classify *K. africana* as primary homothallic non-contiguous (PHN).

Kazachstania rosinii has a *MAT***a**1 gene located between *CAN1* and *RNH203*, similar to most *Kazachstania* species [36], and a *MAT*α1-*MAT*α2 gene pair located between homologs of *HYR1* and a flocculin (*FLO*) gene at a locus that appears to be subtelomeric. Since there are no repeated sequences in the vicinity of the *MAT* genes, we conclude that *K. rosinii* is primary homothallic non-contiguous (PHN).

The *Kazachstania yakushimaensis* assembly contains two 9-kb *MAT* contigs that appear to be alleles. One contains *MAT***a**1, the other contains *MAT*α1-*MAT*α2, and the two contigs are identical over their first 1 kb and last 1 kb. The *K. yakushimaensis MAT* contigs lie between *CAN1* and *RNH203* genes. The allele-specific regions (Y**a** and Yα) are 7 kb, which is unusually long for a Saccharomycetaceae species, but they contain no additional genes. We therefore classified *K. yakushimaensis* as a heterothallic (HET) diploid.

Kazachstania transvaalensis is the closest relative of *K. yakushimaensis.* The *K. transvaalensis* assembly is rather fragmented and this genome has a high content of repeat sequences, but the available data are consistent with *K. transvaalensis* having the same organization as *K. yakushimaensis*, so we classified it as heterothallic (HET) diploid.

In *Lachancea kluyveri*, synteny indicates that both of the silent loci (*HML* and *HMR*) have been lost [21, 22]. The sequenced strain is diploid, and the species is known to be heterothallic [44]. We therefore classified *L. kluyveri* as heterothallic (HET).

In addition to the species mentioned above, 17 more Saccharomycetaceae assemblies have only two or fewer genomic loci containing *MAT* genes, instead of the three expected for a 3LOC species. Many of these assemblies are highly fragmented. In 6 assemblies (*Kazachstania unispora*, *Kazachstania taiaensis*, *Kazachstania bromeliacearum*, *Tetrapisispora fleetii*, *Kluyveromyces aestuari,* and *Kluyveromyces dobzhanskii*), coverage data suggests the existence of additional loci that were collapsed onto one contig. For 9 other species (*Saccharomyces mikatae* [45], *Saccharomyces kudriavzevii* [46], *Saccharomyces arboricola* [47], *Nakaseomyces bacillisporus* [48], *Torulaspora pretoriensis*, *Torulaspora franciscae*, *Torulaspora microellipsoides* [49], *Zygosaccharomyces bailii* [50, 51], and *Eremothecium cymbalariae* [52]), synteny or the presence of X and Z regions suggests that the apparent absence of 3 loci in the assembly used in the Y1000 Project dataset is due to a misassembly rather than a real deletion of one of the loci. For most of these 9 species a standard 3LOC system has been reported in the literature, in some cases from a different strain than was analyzed in the Y1000 dataset. In the final 2 cases (*Lachancea quebecensis* [53] and *Lachancea lanzarotensis* [54]), synteny with their closest relatives (*Lachancea thermotolerans* and *Lachancea fantastica,* respectively [22]) also suggests that the lack of 3 loci is due to misassembly. In all 17 cases, we conclude that the species in question are capable of mating type switching and should be classified as 3LOC.

Saccharomycodaceae

8 species: genus *Hanseniaspora* and species *Kloeckera hatyaiensis*. Four species in this family (*Hanseniaspora uvarum*, *H. pseudoguilliermondii*, *H. valbyensis,* and *H. osmophila*) contain only a *MAT***a** locus or only a *MAT*α locus. We classify them as heterothallic (HET).

Three species (*Kloeckera hatyaiensis*, *Hanseniaspora singularis,* and *Hanseniaspora vineae*) contain both a *MAT***a** and a *MAT*α locus and synteny suggests they are diploid assemblies. *Hanseniaspora clermontiae* contains both a *MAT***a** and a *MAT*α locus in very short contigs. In all 4 cases, we classify these species as diploid assemblies of heterothallic species (HET).

Phaffomycetaceae

34 species: genera *Cyberlindnera, Wickerhamomyces, Phaffomyces, Barnettozyma, Starmera,* and species *Candida mycetangii, Candida freyschussii, Candida orba, Candida montana, Candida stellimalicola,* and *Candida ponderosae.*

Cyberlindnera jadinii and *Cyberlindnera maclurae* each have assemblies of *MAT* loci suggesting they are diploid assemblies of heterothallic species. Diploidy is suggested by the presence of flanking sequences around the loci and, in the case of *C. maclurae*, also by the coverage data.

Cyberlindnera saturnus is one of two species that we infer to be FF1 species whose genome assemblies were affected by an artifact caused by the DISCOVAR assembler. In the *C. saturnus* assembly, a 49 kb (13 gene) region containing *MAT***a** genes (**a**1 and **a**2) at one end and *MAT*α genes (α1 and α2) at the other end is flanked by an inverted repeat (Data S2C), so we conclude that *C. saturnus* can switch mating-types by a flip/flop mechanism using one IR (FF1). In the reported *C. saturnus* assembly [6], the IRs are 118 kb long and differ by only 1 nucleotide, and extend out to the ends of the contig that contains them (NCBI accession PPNR02000017.1). We suspect that the length of the IRs has been artifactually extended by the assembler (DISCOVAR) that was used for this genome, which came from the type strain of *C. saturnus* (NRRL Y-17396). In independent SPAdes assemblies of three other *C. saturnus* strains from the NCYC collection (NCYC22, NCYC23, and NCYC57) we found a similar 49-kb region flanked by short IRs that ran to the end of the SPAdes contigs. Unusually for an FF1 system, the genes *SLA2* and *DIC1* are located inside the invertible region in *C. saturnus* and do not form part of the IR. By synteny with *C. saturnus*, we infer that its close relatives *Cyberlindnera mrakii* and *Cyberlindnera suaveolens* are also FF1 species.

Starmera quercuum is the second species that we infer to be an FF1 species whose genome assembly was affected by an artifact caused by the DISCOVAR assembler (Data S2C). In the *S. quercuum* assembly, the *MAT* contig (NCBI accession PPIB01000006.1) contains a unique 61-kb (21 gene) region with *MAT***a** genes at one end and *MAT*α genes at the other, flanked by IRs that were assembled as 195 kb long and differ by only 1 nucleotide. As with *Cyberlindnera saturnus*, we conclude that *S. quercuum* is an FF1 species with misassembled IRs of unknown length.

Barnettozyma hawaiiensis, *B. populi*, *B. californica,* and *B. salicaria* each have a *MAT* locus assembly suggesting that they are primary homothallic contiguous (PHC). All four canonical *MAT* genes are found next to one another, and in all these species, except *B. populi,* it is in the context of a longer contig, with no repeats present. It is worth noting that another closely related species, *B. pratensis*, shares synteny with these four but has lost its *MAT***a** genes and is classified as heterothallic (HET).

The *MAT* locus of *Wickerhamomyces hampshirensis* suggests it has a primary homothallic contiguous (PHC) arrangement. All four canonical *MAT* genes are found in the middle of a long contig very close to one another with no apparent repeats that would allow the species to flip-flop.

In *Wickerhamomyces canadensis*, *MAT***a** genes are present between full-length *SLA2* and *DIC1* genes in the middle of large (612 kb) contig, and *MAT*α genes are present on a short (7 kb) contig (Data S2D). The *MAT* genes are flanked by two sequences that are repeated on the two contigs: a 1.7 kb repeat containing the 3′ end of *SLA2* (97% DNA sequence identity between the copies), and a 213 bp repeat containing the 5′ end of *DIC1* (100% identity). Both contigs extend beyond these repeats, and in the small contig the available 1 kb of sequence upstream of the truncated *SLA2* appears to include a telomere, with multiple tandem repeats of the 11 bp sequence ATGGTGTTCTG (Data S2D). We interpret these data as indicating that *W. canadensis* is secondary homothallic with an FF2 mechanism and telomeric silencing, because its genomic organization resembles that in *K. phaffii*, although we do not know whether its *MAT***a** and *MAT*α genes are on the same chromosome.

Another possibility is that *W. canadensis* is secondary homothallic but uses a mechanism other than inversion to exchange DNA between the expression locus (between the complete *SLA2* and *DIC1* genes) and the silenced locus (beside the telomere). In any case, the existence of repeats flanking the *W. canadensis MAT* genes shows that it is not primary homothallic. The duplicated *SLA2* region maintains an intact open reading frame, unlike the PHN species we report that have *SLA2* pseudogenes near a subtelomeric *MAT* locus. In the closely related *Wickerhamomyces* sp. NRRL YB-2243 (Data S2D), there is only one set of *MAT* genes, in the arrangement *SLA2*–*MAT*α1–*MAT*α2–ORF–*DIC1*, so *Wickerhamomyces* sp. NRRL YB-2243 is heterothallic and opposite in mating type at the ancestral *MAT* locus to the sequenced *W. canadensis* strain.

The remaining 22 sequenced species in family Phaffomycetaceae either have only *MAT***a** genes or have only *MAT*α genes and we conclude they are heterothallic (HET).

CUG-Ser2 clade

4 species: genera *Ascoidea* and *Saccharomycopsis*. *Ascoidea asiatica* has a *MAT* locus flanked by *ADR1* and *MHP1* (Data S2A). The genes *AGE1* and *STE20* have become integrated into the allele-specific (Y**a** / Yα) regions of its *MAT* locus, similar to the way that extra genes (*PIK*, *PAP*, *OBP*) are integrated into the *MAT* (*MTL*) locus of *Candida albicans* [55]. One of the *A. asiatica MAT* alleles contains *MAT***a**2–*AGE1***a**–*STE20***a**–*MAT***a**1, and the other contains *AGE1*α–*MAT*α1–*STE20*α. We did not find a *MAT*α2 gene in *A. asiatica.* The **a** and α versions of *AGE1* and *STE20* are in different orientations in the two alleles, with approximately 50% amino acid sequence identity in both of the protein pairs. We classify *A. asiatica* as a heterothallic diploid (HET).

Ascoidea rubescens (Data S2A) is a flip-flopping species previously described [7]. It has a single *MAT* region with *MAT***a**1-*MAT***a**2 and *MAT*α1-*MAT*α2 genes separated by 44 kb of non-coding DNA, flanked by 2-kb inverted repeats that include the 5′ end of the *MAT***a**1 gene. One of the copies of the IR is right beside a telomere, so telomeric silencing of transcription is probable. Riley et al. [7] showed that different strains of *A. rubescens* contain the invertible region in different orientations. We classify *A. rubescens* as a flip-flopper with one set of inverted repeats (FF1).

Comparison of the two *Ascoidea* species *A. rubescens* (FF1) and *A. asiatica* (HET) reveals some details of how flip/flop switching originated in this genus (Data S2A). In the derived FF1 organization seen in *A. rubescens*, the *MAT* genes have moved about 200 kb from the *ADR1-AGE1-MHP1* region and are now located beside a telomere and flanked by an IR (Data S2A). Moreover, *STE20* has been lost from the *A. rubescens* genome, and its role as a mating-specific PAK kinase may have been taken over by an *A. rubescens*-specific duplication of the kinase *CLA4*. The single *AGE1* gene of *A. rubescens* appears to be derived from an *AGE1***a** gene, because it is more closely related to *A. asiatica*'s *AGE1***a** than its *AGE1*α, and in the same orientation relative to *MHP1* (Data S2A).

Saccharomycopsis capsularis has *MAT*α1 and *MAT*α2 genes located between *SLA2* and *YJR098C* in the middle of a large (398 kb) contig, and *MAT***a**1 and *MAT***a**2 beside a *SLA2* pseudogene near one end of a short contig (27 kb) that contains almost no other intact protein-coding genes, from which we conclude that *S. capsularis* is primary homothallic (PHN). *Saccharomycopsis malanga* has only a *MAT***a**2 gene, located between *SLA2* and *YJR098C*, and we classify it as heterothallic (HET).

CUG-Ser1 clade

93 species: genera *Metschnikowia, Hyphopichia, Danielozyma, Lodderomyces, Spathaspora, Scheffersomyces, Suhomyces, Kodamaea, Aciculoconidium, Teunomyces, Wickerhamia, Priceomyces, Debaryomyces, Millerozyma, Yamadazyma, Meyerozyma, Kurtzmaniella, Cephaloascus, Babjeviella*, and 28 species of the genus *Candida.*

Two species, both in the CUG-Ser1 clade, were classified as NOMAT because we could not identify any of the four canonical *MAT* genes in their genomes. These species are *Lodderomyces elongisporus* and *Candida sojae*. The *L. elongisporus* genome was Sanger sequenced and assembled into 11 large scaffolds (145 contigs; contig N_{50} = 261 kb; accession number AAPO01000000.1) by Butler et al. [18], who reported that there are no *MAT* genes in the sequenced strain. In addition, multiple other *L. elongisporus* isolates were examined by PCR and sequencing, but no *MAT* genes were identified in them [18, 19]. The *Candida sojae* genome was sequenced by Borelli et al. [20], accession number LMTL00000000.1 (48x coverage Illumina; contig N_{50} = 56 kb). Using query sequences from its close relative *C. tropicalis*, we were unable to find any *MAT* genes in the *C. sojae* assembly, and we were also unable to find many of the genes that are normally near the *MAT* locus in CUG-Ser1 clade species such as *orf19.3202*, *PAP1*, *RCY1*, and *orf19.3204*. The sequenced strain may therefore have lost a large region of DNA spanning the *MAT* locus.

Spathaspora passalidarum is a species previously discussed in the literature [7, 17]. It has contiguous *MAT* genes of both types (**a**1, α1 and α2) close to one another with no repeats indicating the ability to switch mating type. We classify it as a member of the PHC category. (The *MAT***a**1 of *S. passalidarum* was overlooked in [7], leading to an incorrect classification as heterothallic. This error was corrected in [17], leading to recognition as PHC.) The assemblies of *Spathaspora hagerdaliae* and *Spathaspora gorwiae* contain both *MAT***a** and *MAT*α genes, but they are highly fragmented, and we classify them as heterothallic diploids (HET) as there is no clear evidence for homothallism in these species. Another species from the genus, *Spathaspora arboriae*, has *MAT***a**1 and *MAT***a**2 genes but no *MAT*α genes [17] and is therefore heterothallic (HET). *Spathaspora girioi* is syntenic with *S. arboriae* at the *MAT***a** locus, and its *MAT*α sequence seems to be allelic to *MAT***a**, so we infer that *S. girioi* is a heterothallic diploid (HET).

Scheffersomyces stipitis has a single *MAT* locus with contiguous *MAT***a**1, *MAT***a**2 and *MAT*α1 genes close to each other [17] and no repeats indicating the ability to switch mating type, so we characterize it as primary homothallic contiguous (PHC).

In *Wickerhamia fluorescens*, *MAT*α genes are located near *PIK1*, *PAP1*, and *OBP* genes, as in other members of the CUG-Ser1 clade. However, it also contains *MAT***a** genes 220 kb away, at a site that is probably subtelomeric (33 kb from one end of a 1.6 Mb scaffold), so we classified this species as PHN.

Candida tropicalis has *MAT***a** and *MAT*α genes on separate contigs [17]. Repeated sequences at the ends of them indicate that they are allelic. We conclude that it is a diploid assembly of a heterothallic species (HET).

The entire *Priceomyces* genus including *P. haplophilus*, *P. castillae*, *P. medius* and *P. carsonii* has a single *MAT* locus with contiguous *MAT***a** and *MAT*α genes close to one another and shared synteny. We infer that the genus is primary homothallic contiguous (PHC).

The *MAT* locus of *Debaryomyces hansenii* was previously described [17] and contains neighboring *MAT***a**1, *MAT***a**2 and *MAT*α1 genes. The five other sequenced *Debaryomyces* species (*D. maramus*, *D. nepalensis*, *D. prosopidis*, *D. fabryi* and *D. subglobosus*) all have *MAT* loci syntenic with *D. hansenii*, and with the same gene content. We classify all these species as members of the PHC category.

Two monophyletic species of the genus *Yamadazyma* (*Y. nakazawae* and *Y. philogaea*) have non-contiguous *MAT***a** and *MAT*α loci that are non-allelic. They both have *MAT*α1 and *MAT*α2 genes located near *PIK1*, *PAP1*, and *OBP* genes similar to other members of the CUG-Ser1 clade, but they also have *MAT***a**1 and *MAT***a**2 genes. The *MAT***a** genes are located at the ends of large contigs (142 kb in *Y. nakazawae*; 215 kb in *Y. philogaea*), and are close to homologs of the gene CANTEDRAFT_101864, which in the closely related species *Yamadazyma tenuis* (formerly called *Candida tenuis*) is a member of a subtelomeric gene family [56]. Because there are no repeat sequences near the *MAT* genes of *Y. nakazawae* and *Y. philogaea*, there is no evidence that they can switch mating type, and we conclude they are primary homothallic with non-contiguous *MAT* genes (PHN).

Cephaloascus fragrans has a single *MAT* locus with contiguous *MAT***a** and *MAT*α genes close to one another. We conclude that it is primary homothallic contiguous (PHC).

Pichiaceae

61 species: genera *Ambrosiozyma, Brettanomyces, Citeromyces, Dekkera, Komagataella, Kregervanrija, Kuraishia, Martiniozyma, Ogataea, Pichia, Saturnispora* and species *Candida arabinofermentans, Candida boidinii, Candida sorboxylosa*, and *Candida succiphila.*

The *Pichia kudriavzevii* genome in the Y1000 Project dataset is misassembled at the *MAT* locus. From our laboratory's reference genome sequence for this species [57], which is diploid and has both alleles of the *MAT* locus, we deduce that it is a heterothallic species (HET).

Pichia nakasei, *Pichia occidentalis*, *Pichia membranifaciens,* and *Candida sorboxylosa* all have assemblies with two *MAT* loci with flanking sequences indicating that they are diploid assemblies of heterothallic species (HET).

The assemblies of *MAT* regions of *Saturnispora* species are fairly fragmented. By manually merging a few contigs of the assembly of *Saturnispora zaruensis* (Figure 3B), we conclude that it is a flip-flopper with two sets of repeats (inner and outer) around its two *MAT* loci (FF2). There is a centromere in the region between the two *MAT* loci, which is an arrangement similar to that of *Komagataella phaffii* [13, 27] (Figure 3A). However, *S. zaruensis* differs from *K. phaffii* in that (i) the centromere is very close to one set of *MAT* genes, and (ii) none of the *MAT* genes are close to a telomere. We propose that proximity to the centromere is the mechanism used by *S. zaruensis* to repress transcription of the silent *MAT* locus, and that the entire 194-kb region between the two *MAT* loci inverts during switching, by recombination between the inner pair of repeats. Under this hypothesis, the inner IR is necessary for switching, while the function of the outer IR remains unknown, which is the opposite of what we know about the IRs in *K. phaffii* [13]. The inference that there is a centromere near the end of the 194-kb region is based on the observations that the gene arrangement *VPS36 – HYM1 – CEN – SLA2_fragment – MAT* flanks a centromere in *Ogataea polymorpha* (cf. Figure 3B), and that the putative *CEN* region in *S. zaruensis* contains a pseudogene of a Ty5-like retroelement, which is centromere-associated in many species [13].

Saturnispora hagleri, *Saturnispora dispora,* and *Saturnispora serradocipensis*, despite multiple breaks in their assemblies, clearly share synteny with *S. zaruensis*, so we conclude they are also flip-floppers with two sets of repeats (FF2).

Saturnispora mendoncae and *Martiniozyma abiesophila* both have two *MAT* loci (**a** and α), that look allelic and share synteny with *Pichia kudriavzevii*. We conclude they are diploid assemblies of heterothallic species (HET).

The published assembly of the *Kregervanrija fluxuum* genome [6] was assembled using the SPAdes assember. It has a single *MAT* scaffold with *MAT***a**1-*MAT***a**2 and *MAT*α1-*MAT*α2 genes, respectively, at opposite ends of a 12 kb contig that contains no other genes. No repeats are present. However, in an independent DISCOVAR assembly of the same *Kregervanrija fluxuum* Illumina data, the 12 kb region is flanked by two identical 15-kb sequences that form a large IR extending to the ends of the contig (Data S2C). This situation resembles the DISCOVAR assemblies of the *Cyberlindnera saturnus* and *Starmera quercuum* genomes, and we believe that, in each case, an IR is present (i.e., it is an FF1 species) but the length of the IR has been artifactually inflated by DISCOVAR.

Kregervanrija delftensis (SPAdes assembly) has an identical *MAT* locus organization to *K. fluxuum*. We conclude that *K. delftensis,* and *K. fluxuum* are both FF1 secondary homothallic species.

Ambrosiozyma ambrosiae and *A. philentoma* each have two *MAT* loci (**a** and α) that are not contiguous. In both species, the *MAT***a** genes are in the ancestral context *SLA2–MAT***a**1*–MAT***a**2*–DIC1–ASA1*, and the *MAT*α genes are in the non-ancestral context *YLR001C–IZH3–MAT*α1*–MAT*α2*–EFM5*. There are no flanking sequences that would indicate that they are allelic, and no repeat sequences that would indicate a mating-type switching mechanism. Both of the *MAT* loci in both species are in the middle of large contigs and therefore not subtelomeric. There is a large gene-free region between the *MAT*α genes and the next gene, *IZH3* (11 kb in *A. ambrosiae*, 19 kb in *A. philentoma*). In *Saccharomyces cerevisiae*, *YLR001C* and *EFM5* are genes located immediately next to centromeres. We infer that *A. ambrosiae* and *A. philentoma* are primary homothallic non-contiguous (PHN).

Ambrosiozyma oregonensis has two *MAT* loci (**a** and α) on very short contigs with flanking repeated sequences around them. We infer that it is a diploid assembly of a heterothallic species (HET).

Brettanomyces anomalus has two *MAT* loci that have flanking sequences suggesting they are alleles of each other. We conclude that it is a diploid assembly of a heterothallic species (HET).

Only 5 species in the *Ogataea* genus clade were classified as heterothallic (HET): *O. methylivora, O. ramenticola, Candida succiphila, O. nitratoaversa,* and *Candida arabinofermentans.*

Ogataea naganishii (PHC) has the gene organization *SLA2–MAT***a**1*–MAT***a**2*–MAT*α1*–MAT*α2*–DIC1*. There no gaps in the assembly in this region, and no repeated sequences that would suggest the ability to flip-flop. The *MAT***a**2 and *MAT*α1 genes are separated by 7 kb of noncoding DNA. We conclude that it is primary homothallic contiguous (PHC).

12-species *Ogataea* FF1 clade: *Ogataea polymorpha*, *O. parapolymorpha,* and *O. nonfermentans* have contiguous *MAT***a** and *MAT*α genes in a flip-flop-like arrangement with an inverted repeat (part of the *SLA2* gene) at the ends. We infer they are secondary homothallic flip-floppers with a single IR (FF1). Switching by inversion in *O. polymorpha* has been reported [13, 14]. *Ogataea philodendri*, *O. kodamae*, *O. minuta*, *O. henricii*, *O. pini*, *O. glucozyma*, *O. zsoltii*, *O. trehaloabstinens,* and *O. populialbae* have broken assemblies at their *MAT* loci, but they share synteny with *Ogataea polymorpha* and we conclude they are also secondary homothallic flip-floppers with a single IR (FF1). Switching by inversion in *O. minuta* has been reported [26].

Ogataea trehalophila and *O. methanolica* form a pair of sister FF1 species. They each have non-contiguous *MAT***a** and *MAT*α loci, that are flanked by a repeated sequence derived from *SLA2*. We infer that these two species also use a flop/flop switching mechanism (FF1), but the invertible region appears to be much larger than in *O. polymorpha* (several hundred kb). The *O. methanolica* assembly contains two very large duplications, one of a 23-kb region that ends at *SLA2*, and one of an 8-kb region, but we suspect that the length of these duplications may be artifactually inflated, because the *O. methanolica* genome was sequenced using a library with DNA inserts approximately 3 kb long [6], so in principle it is unlikely that identical repeat sequences, 23 kb or 8 kb long, as are present in the assembly (AbySS assembler), were correctly resolved.

Ogataea pilisensis (FF1) has a 7-kb contig that contains genes *SLA2-SUI1-CWC25* and is present at 2x sequence coverage compared to the rest of the assembly. The 3′ end of *SLA2* overlaps with the ends of two other contigs, one containing *MAT*α1-*MAT*α2 and the other containing *MAT***a**1-*MAT***a**2. We therefore classified *O. pilisensis* as a secondary homothallic (FF1) with a 7-kb IR. *Ogataea nitratoaversa* and *Candida arabinofermentans* are closely related to *O. pilisensis* and show conserved synteny with it at the *MAT* locus, but they both appear to be heterothallic (HET). *O. nitratoaversa* contains only *MAT*α1-α2 genes, and *C. arabinofermentans* contains only *MAT***a**1-**a**2 genes [7], located in both cases between *SLA2* and *DIC1*.

The FF1 species of *Ogataea* described above form three distinct clades within the genus. There are two equally parsimonious hypotheses about the transitions that led to this situation (either three independent gains of FF1, or one ancestral gain, followed by two losses). We conclude that three gains of FF1 is a more probable explanation, based on the gene orders that exist at the *MAT* loci. The main reason is that the region between the IRs is very different (in size and endpoints) among the three FF1 clades of *Ogataea*, so if the whole genus *Ogataea* was ancestrally FF1, it would be necessary to also postulate multiple reorganizations of the FF1 system within the genus. Specifically:

In the *O. polymorpha* FF1 group (12 species) the invertible region between the IRs is only 19 kb long, with *DIC1* at one end and *TPK3* near the other end.

In the pair of FF1 species *O. trehalophila* and *O. methanolica*, the invertible region is much larger (> 416 kb and > 700 kb, respectively), with a *GCN4*-like gene at one end and *REC102* at the other end, in both species, and an IR that terminates in *SLA2*. *Candida arabinofermentans* is a heterothallic outgroup to these two species, but the *C. arabinofermentans MAT* locus is found in the context *SLA2-MAT-DIC1* which is an ancestral and commonly observed context. If the heterothallism of *C. arabinofermentans* was a derived situation (i.e., an FF1 → HET transition from an ancestor resembling *O. trehalophila*/*O. methanolica*), we would have expected its *MAT* locus to be in the context *SLA2-MAT-GCN4* or *SLA2-MAT-REC102*. It is more parsimonious to propose that the *C. arabinofermentans* arrangement is ancestral, and the *O. trehalophila*/*O. methanolica* arrangement is derived (i.e., a HET → FF1 transition).

Similarly, the FF1 species *O. pilisensis* has a large invertible region of at least 159 kb, with *SLA2-MAT***a**-*DIC1* at one end and *SLA2-MAT*α-*LRS4* at the other, where *SLA2* forms part of the IR. Its sister heterothallic species *O. nitratoaversa* has the ancestral context *SLA2-MAT-DIC1*. Since the arrangement *SLA2-MAT-LRS4* is not seen in any other species, either (i) this arrangement originated *de novo* when an FF1 system emerged in *O. pilisensis*, after it had diverged from *O. nitratoaversa* (i.e., HET → FF1 transition); or (ii) this arrangement originated in *O. pilisensis* by reorganization of one endpoint of an existing FF1 system, and separately *O. nitratoaversa* lost one of its two *MAT* loci, which happened to be the one at the non-ancestral location (i.e., FF1 → HET transition). We consider the first scenario to be more parsimonious.

Kuraishia molischiana has a *MAT*α locus that is syntenic with the single *MAT* locus of the heterothallic species *K. capsulata*, but *K. molisciana* also has a *MAT***a**1 at one end of a large (458 kb) contig. The *MAT***a**1 gene is located beside a badly degraded pseudogene of *SLA2*. There are no repeats around the *MAT* genes, so we infer that *K. molisciana* is primary homothallic with non-contiguous *MAT* loci (PHN).

Citeromyces matritensis has two non-contiguous *MAT* loci (**a** and α) with flanking sequences suggesting that it is a diploid assembly of a heterothallic species (HET). *Citeromyces hawaiiensis* partly shares synteny with it, but is missing the flanking sequences, possibly due to the contigs being broken at them. *Citeromyces siamensis* has a more fragmented assembly at the *MAT* loci. We concluded that *Citeromyces hawaiiensis* and *C. siamensis* are both also diploid assemblies of heterothallic species (HET).

Komagataella phaffii (formerly called *Pichia pastoris*) is a flip-flopping secondary homothallic species with two sets of inverted repeats (FF2) around both the *MAT***a** and the *MAT*α genes [13] (Figure 3A). The invertible region is approximately 138 kb. The assemblies of *Komagataella pseudopastoris* and *K. populi* are broken around their two *MAT* loci, but flanking repeats are present. From their phylogenetic relationship with *K. phaffii,* we infer they are also secondary homothallic species using the flip-flopping system with two sets of inverted repeats (FF2).

CUG-Ala clade

5 species: genera *Peterozyma* and *Nakazawaea*, and species *Pachysolen tannophilus*. *Peterozyma xylosa* and *Peterozyma toletana* (Data S2B) were classified as primary homothallic non-contiguous (PHN). Both of these species contain both *MAT*α and *MAT***a** genes, at non-contiguous sites. Their *MAT*α genes are located between *SLA2* and *SPC3*, syntenic with the *MAT* loci of several heterothallic Pichiaceae species. These *MAT*α genes are approximately 100 kb from the rDNA array. The *MAT***a** genes are present on the opposite side of the rDNA array, immediately beside the rDNA. We classified these *Peterozyma* species as PHN because there are no repeat sequences that could catalyze exchange between the *MAT*α and *MAT***a** loci.

Nakazawaea peltata and *Nakazawaea holstii* both have only a *MAT*α locus. We conclude that they are heterothallic (HET).

Pachysolen tannophilus is a species whose *MAT* locus was previously described [7]. It has a single invertible 8 kb-long *MAT* region with *MAT***a** and *MAT*α genes at its ends, flanked by 2-kb inverted repeats (Data S2B). Riley et al. [7] experimentally confirmed that the region can be induced to invert by growth of a culture of *P. tannophilus* in nitrogen-depleted media. However, it is unclear how orientation-specific silencing of one of the two *MAT* gene types is achieved in this species [7, 17]. We classify it as a member of the FF1 category.

Sporopachydermia

2 species: *Sporopachydermia quercuum* and *S. lactativora*. *Sporopachydermia quercuum* has contiguous *MAT***a** and *MAT*α genes very close to each other and does not have inverted repeats around them. We classify it as primary homothallic contiguous (PHC). *Sporopachydermia lactativora* has only *MAT***a** genes and therefore is heterothallic (HET).

Dipodascaceae/Trichomonascaceae

37 species: genera *Arxula, Blastobotrys, Deakozyma, Diddensiella, Dipodascus, Nadsonia, Geotrichum, Groenewaldozyma, Magnusiomyces, Middelhovenomyces, Saprochaete, Spencermartinsiella, Starmerella, Sugiyamaella, Wickerhamiella, Yarrowia, Zygoascus*; and species *Candida hispaniensis* and *Candida incommunis.*

Starmerella bombicola is classified as HET. The genome of the type strain of this species (JCM 9596 / NRRL Y-17069 / NBRC 10243) has been sequenced three times, including a high contiguity genome sequence by RIKEN (contig N_{50} = 2.9 Mb) [6]. It contains an ORF with no identifiable domains, in the context *SLA2-ORF-TFC1.* The corresponding genomic region in two closely related species (*Candida apicola* and *Wickerhamiella domercqiae*) contains the series of genes *SLA2-MATα*1*-MFα-TFC1*, where *MFα* is the gene for alpha-pheromone (which is not commonly present at the *MAT* locus of ascomycetes). Similarly, a second strain of *S. bombicola* (PYCC 5882) contains the series *SLA2-MATα*1*-MFα* at the end of one contig (PEOC01000491 [58]), though *TFC1* is elsewhere in the genome. It is therefore possible that the ORF in *S. bombicola* codes for a determinant of *MAT***a** mating type, but we are unable to detect any sequence relationship between it and known **a**1 or **a**2 proteins. We classified *S. bombicola* as heterothallic (HET) on the basis of the *MATα*1 gene present in strain PYCC 5882.

Blastobotrys proliferans has a *MATα*1 gene located between full-length *SLA2* and *APN2* genes, syntenic with the single *MAT* locus in other *Blastobotrys* species such as *B. adeninivorans* [59]. The *MATα*2 gene is absent throughout this genus. However, *B. proliferans* also has a *MAT***a**2 gene, located between pseudogenes of *SLA2* and *APN2*, at a site that appears to be subtelomeric. The site is at the end of a 280 kb contig, and near a homolog of the *S. cerevisiae* gene *SGS1*, which is repeated on the subtelomeric regions of the well-assembled *B. adeninivorans* genome [59]. We infer that *B. proliferans* is primary homothallic non-contiguous (PHN).

Nadsonia fulvescens var. *elongata* has separate *MAT***a** and *MATα* loci that are not allelic, and there are no repeats that would constitute evidence of the ability to switch mating type. The *MAT***a** genes are near a telomere (Data S2E). We conclude that it is primary homothallic non-contiguous (PHN).

Trigonopsidaceae

6 species: genera *Botryozyma*, *Tortispora* and *Trigonopsis*. The genome assembly of *Botryozyma nematodophila* consists of 17.3 Mb in almost 11,000 scaffolds with average nuclear coverage only 4x and N_{50} = 2575 bp. This high level of fragmentation makes synteny analysis impossible. However, we detected part of a *MATα*2 gene downstream of the 3′ end of *SLA2* in this species, so we tentatively classified *B. nematodophila* as heterothallic (HET).

Tortispora ganteri has *MAT***a** and *MATα* genes on contigs with repeated flanking sequences, suggesting that these are allelic. We conclude that it is a diploid assembly of a heterothallic species (HET). The two other *Tortispora* species studied were also classified as haploid heterothallics (HET), with only *MATα* genes in *T. caseinolytica* [7], and only *MAT***a** genes in *T. starmeri*.

Trigonopsis variabilis has only *MAT***a** genes, and *Trigonopsis vinaria* has only *MATα* genes, so we classified these two species as heterothallic (HET).

Lipomycetacae

9 species: genus *Lipomyces*. The *MAT* loci of all the sequenced species in the genus *Lipomyces* are illustrated in Data S2F and discussed below. In addition to the canonical *MAT* genes, some *Lipomyces* species have an extra gene at their *MAT* locus, coding for an HMG domain protein (i.e., a DNA-binding protein distantly related to the **a**2 and α1 proteins) whose function is unknown [7]. We refer to this gene here as *HMGX*. It is present in 6 of the 9 sequenced *Lipomyces* species (Data S2F).

Lipomyces starkeyi, *L. arxii*, *L. mesembrius*, and *L. kononenkoae* form a 4-species clade that we classified as PHC. Each of them has all four canonical *MAT* genes as direct neighbors of one another at a single genomic site (Data S2F). This site appears to be the ancestral *MAT* site, as it is situated between the gene *SLA2* on one side and *APN2* and *MLH3* on the other. These four species have the *HMGX* gene, whose location in the *MAT* locus is conserved among *L. starkeyi*, *L. arxi* and *L. mesembrius*. However, in *L. kononenkoae HMGX* is at a different location in the genome, away from the *MAT* region. For all four species we conclude that they belong in the PHC category.

The fairly fragmented assembly of *Lipomyces doorenjongii* (Data S2F) has two short contigs, containing a *MAT***a** locus (**a**1 and **a**2 genes) and a *MATα* locus (α1 and α2 genes) respectively. By manually identifying their overlaps with other contigs, we infer they are parts of a diploid assembly, and that *L. doorenjongii* is a heterothallic diploid whose *MAT* locus is at the ancestral location between *SLA2* and *MAM3* on one side, and *MLH3* on the other. Coverage data show that one of the contigs (NODE_122) has double coverage and can also be connected to a different contig (NODE_120), which is not ancestrally at a *MAT*-related position (Data S2F). The presence of NODE_120 raises the possibility that there could be a second *MAT*-like locus in the genome. Nevertheless, we decided to classify this species as HET (rather than PHN), making it the only heterothallic genome in the genus *Lipomyces* and one of the few (three) examples of an evolutionary transition toward heterothallism. This decision is conservative, because if *L. doorenjongii* is PHN, the ratio between transitions toward and away from homothallism would be 31:2 rather than the 31:3 ratio we report.

Lipomyces japonicus has two sets of *MAT* genes, one located at the ancestral position (between *SLA2* and *APN2–MLH3*), and the other located on a different contig (Data S2F). The two *MAT* loci must be at least 28 kb apart in the genome. The ancestral site contains *MAT***a**1, *MAT***a**2, and *HMGX*, while the non-ancestral site contains *MATα*1, *MATα*2 and another *HMGX* gene (46% amino acid sequence identity between the two *HMGX* genes). The contig (23 kb) containing the non-ancestral site contains no other genes apart

from the *MAT*α genes and *HMGX*, except for some pseudogenes of retrotransposons and transposases, so it may be heterochromatic but we have no indication that it is subtelomeric. We classify *L. japonicus* as a member of the PHN category.

Lipomyces lipofer has a single *MAT* site containing *MAT***a**1, *MAT***a**2, *MAT*α1, *MAT*α2, and a homolog of *HMGX*. Although both *SLA2* and *APN2* are located at other genomic loci, *MLH3* is present in the neighborhood of the *MAT* genes, and so are some other genes (for example an RNA-dependent RNA polymerase (RdRP) on one side and *FOL3* on the other) associated with the ancestral *MAT* locus. We classify this species as a member of the PHC category.

Lipomyces suomiensis has two *MAT* loci. One is at the ancestral location, between *MLH3* and *SLA2* on one side and *APN2* on the other, and contains *MAT***a**1 and *MAT***a**2. The second *MAT* location contains *MAT*α1 and *MAT*α2 flanked by pseudogenes of *SLA2* and *APN2*. We infer that this second location is subtelomeric because the *MAT*α genes are 15 kb from one end of a contig, which terminates in multiple tandem copies of the sequence $(TTTTAGTAGGG)_n$ which resembles known yeast telomeres [60] and also occurs at the ends of several very large contigs in the *L. suomiensis* assembly. The *MAT*α genes are in the center of a 27 kb region that contains no other intact genes. *L. suomiensis* has no HMGX genes. We conclude that *L. suomiensis* belongs in the PHN category.

L. oligophaga forms a clade with *L. suomiensis* and also has two *MAT* loci, one ancestral and one subtelomeric. However, the genotypes of the two *MAT* loci in *L. oligophaga* are the opposites of those in *L. suomiensis* (Data S2F). In *L. oligophaga,* the ancestral *MAT* locus has the arrangement *MLH3–SLA2–MAT*α*1–APN2.* We were unable to detect any *MAT*α2 gene in this species. The second *MAT* locus in *L. oligophaga*, containing *MAT***a**1 and *MAT***a**2 genes, is at one end of a 547 kb contig that terminates in multiple tandem copies of a telomeric repeat $(TTTGAGGG)_n$ which also occurs at the ends of other large contigs in this species. The distance between the end of the *MAT***a**2 gene and the first telomeric repeat is only 47 bp. *L. oligophaga* has no HMGX genes. We conclude that *L. oligophaga* belongs in the PHN category.

We infer that the genus *Lipomyces* is ancestrally PHC, and that there have been two transitions to PHN, and one to HET, in this genus as explained below.

Because *L. lipofer* and the 4-species *L. starkeyi* clade have all four canonical *MAT* genes (**a**1, **a**2, α1, α2) present at the ancestral *MAT* site, we infer that the most recent ancestor of the 7-species clade that includes these five also had this gene arrangement, and therefore would have been classified as PHC. Therefore, we infer a PHC → PHN transition in *L. japonicus*, and a PHC → HET transition in *L. doorenjongii*.

We hypothesize that it may be easier to undergo a transition from PHC to PHN than the other way round, because the situation in which some genes are removed from the ancestral *MAT* locus to some other part of the genome is more likely than one in which genes from two separated *MAT* loci become co-localized at a single site (the ancestral one). Another reason is that we already see such a transition in *L. japonicus*. Therefore we classify the ancestor of the *Lipomyces* genus as PHC, and infer a PHC → PHN transition in the common ancestor of *L. oligophaga* and *L. suomiensis*. However, *L. oligophaga* and *L. suomiensis* have opposite alleles at the ancestral *MAT* locus, so it is possible that each of them represents a separate PHC → PHN transition, but for reasons of parsimony we inferred a single transition in the common ancestor of this pair of species.

QUANTIFICATION AND STATISTICAL ANALYSIS

The data analyses reported in this manuscript do not use any statistical tests.

DATA AND CODE AVAILABILITY

Automated and manual sequence similarity searches were performed using TBLASTN [40]. The Python code used for automating the searches is available at GitHub at https://github.com/tadekkr/Multiple-reinventions-of-mating-type-switching-during-budding-yeast-evolution. Phylogenetic trees in Data S2 were made using Seaview [41].

Current Biology, Volume *29*

Supplemental Information

Multiple Reinventions of Mating-type Switching during Budding Yeast Evolution

Tadeusz Krassowski, Jacek Kominek, Xing-Xing Shen, Dana A. Opulente, Xiaofan Zhou, Antonis Rokas, Chris Todd Hittinger, and Kenneth H. Wolfe

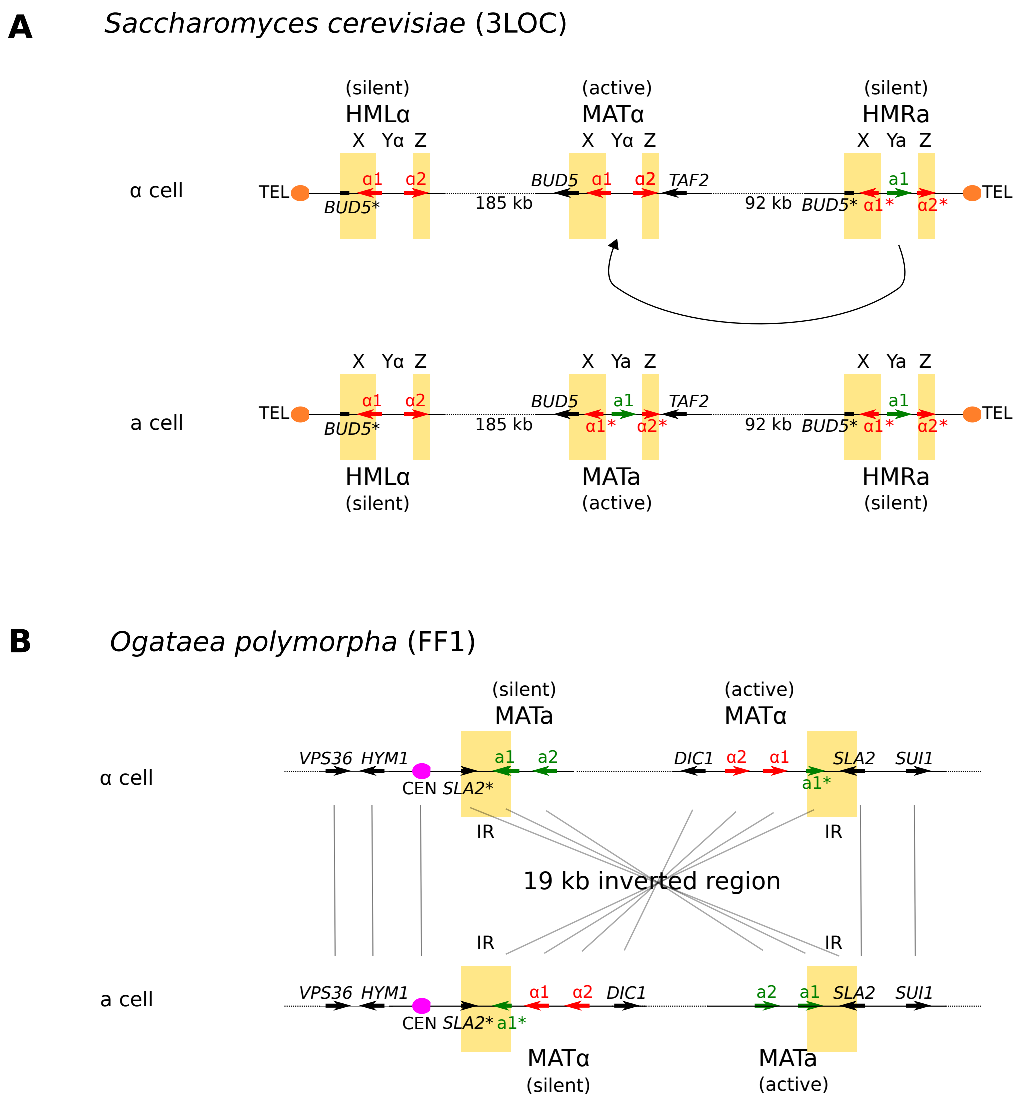

Figure S1. Genomic organization of MAT loci and mating-type switching in *Saccharomyces cerevisiae* and *Ogataea polymorpha*. Related to Figure 1.
Yellow boxes indicate repeats.
